# Supplementary material for: Topographically-controlled contribution of avalanches to glacier mass balance in the 21st century
Source: Nat Commun. 2025 Nov 26;16:10122. doi: 10.1038/s41467-025-65608-z (PMC12657945; doi:10.1038/s41467-025-65608-z)
Supplement: Supplementary file 1 — Supplementary Information [file 41467_2025_65608_MOESM1_ESM.pdf]

Supplementary information for ‘Topographically-controlled contribution of avalanches to glacier accumulation in the 21st century’

Marin Kneib (kneibm@ethz.ch)[1, 2, 3, 4,], Fabien Maussion[4, 5], Fanny Brun[3], Guillem Carcanade[3], Daniel Farinotti[1, 2], Matthias Huss[1, 2, 6], Marit van Tiel[1, 2], Achille Jouberton[7], Patrick Schmitt[4], Lilian Schuster[4], Amaury Dehecq[3], Nicolas Champollion[3]*

1. *Laboratory of Hydraulics, Hydrology and Glaciology (VAW), ETH Zurich, Zurich, Switzerland*
2. *Swiss Federal Institute for Forest, Snow and Landscape Research (WSL), bâtiment ALPOLE, Sion, Switzerland*
3. *Institut des Géosciences de l'Environnement, Université Grenoble Alpes, CNRS, IRD, Grenoble, France*
4. *Department of Atmospheric and Cryospheric Sciences, University of Innsbruck, Innsbruck, Austria*
5. *School of Geographical Sciences, University of Bristol, Bristol, UK*
6. *Department of Geosciences, University of Fribourg, Fribourg, Switzerland*
7. *Institute of Science and Technology Austria, ISTA, Klosterneuburg, Austria*

Supplementary Tables 2
Supplementary Figures 5
Glacier-wide avalanche contribution and glacier variables 5
Influence of avalanches on the recalibration of the mass balance model 8
Altitudinal mass balance profiles 10
Projected ice volume changes 11

Projected avalanche contribution changes 29
Influence of temporarily updating the avalanche contribution 37
Influence of DEM spatial resolution 39
Evaluation against Sentinel-1 avalanche deposits 43
Evaluation against mass balance measurements 44
References 46

Supplementary Tables

Supplementary Table 1: regional avalanche contribution metrics averaged over the period 01/2000 - 12/2019. *These values were used to compute Figure 2.*

RGI region	Regional positive avalanche contribution (%)	Regional negative avalanche contribution (%)	Regional net avalanche contribution (%)	Median avalanche contribution (%)	20th percentile of avalanche contribution (%)	80th percentile of avalanche contribution (%)	Glacier area (km^2)	Total snow accumulation (m w.eq. yr^{-1})
Alaska	5.2	-0.9	4.3	1.4	-0.4	11.9	86,492	2.5
Western Canada and USA	6.8	-1.5	5.2	2.4	-0.2	16.2	14,567	2.3
Arctic Canada North	0.0	-0.1	-0.1	-0.1	-0.2	0.1	105,253	0.7
Arctic Canada South	0.5	-0.2	0.3	0.0	-0.3	0.5	40,760	0.9
Greenland Periphery	2.4	-0.6	1.8	0.3	-0.3	6.6	74,766	1.5
Iceland	0.2	-0.1	0.1	0.2	-0.2	1.8	11,015	2.9
Svalbard and Jan Mayen	0.2	-0.1	0.1	0.1	-0.2	0.7	34,017	1.4
Scandinavia	3.4	-0.6	2.8	0.6	-0.3	9.5	2,958	2.4
Russian Arctic	0.0	-0.1	-0.1	-0.1	-0.2	0.1	52,020	1.0
North Asia	3.2	-1.0	2.1	0.8	-0.3	6.0	2,330	1.3
Central Europe	11.1	-1.9	9.1	4.8	-0.3	26.0	2,044	2.4
Caucasus and Middle East	11.1	-3.7	7.4	3.0	-1.5	15.6	1,139	2.5
Central Asia	3.0	-1.3	4.3	0.2	-0.3	1.6	49,344	1.0
South Asia West	5.9	-1.2	4.7	0.6	-0.5	5.7	33,669	1.2
South Asia East	19.2	-8.0	11.2	2.0	-0.7	15.6	14,492	2.1
Low Latitudes	5.7	-8.0	-2.3	-0.3	-5.9	2.6	2,251	1.9
Southern Andes	2.9	-4.4	-1.5	0.1	-1.7	2.1	29,137	1.5

New Zealand	21.8	-7.2	14.6	2.9	-9.3	30.8	1,125	3.4
Subantarctic and Antarctic Islands	0.5	-0.5	0.0	-0.0	-0.8	1.4	135,762	1.7
Global	3.0	-1.1	2.0	0.4	-0.4	5.9	693,150	1.5

Supplementary Table 2: Statistics of number and area of successfully modelled glaciers for each RGI region.

RGI region	Total number of glaciers	Total glacier area in 2000 (km^2)	Successfully modelled glaciers (%)	Successfully modelled area (%)
01	27108	86725	99.6	100.0
02	18855	14524	98.3	99.9
03	4556	105111	99.4	100.0
04	7415	40888	99.5	100.0
05	18306	89717	99.1	83.3
06	568	11060	100.0	100.0
07	1615	33959	100.0	100.0
08	3417	2949	99.6	100.0
09	1069	51592	99.7	100.0
10	5151	2410	96.5	96.3
11	3927	2092	96.5	99.8
12	1888	1307	80.7	87.9
13	54429	49303	99.3	99.8
14	27988	33568	99.6	99.9
15	13119	14734	99.1	99.7
16	2939	2341	94.2	97.3
17	15908	29429	98.6	99.1
18	3537	1162	97.1	93.9
19	2752	132867	80.4	99.8
Global	214547	705738	98.6	97.7

Supplementary Table 3: Effect of DEM spatial resolution on regional avalanche contribution. *Regional avalanche contribution metrics for Central Europe over the period 01/2000 - 12/2019 for the model run at 50 m, 100 m and variable spatial resolution.*

DEM spatial resolution (m)	50	100	Variable (reference)
Regional net avalanche contribution (%)	9.4	8.9	9.1
Regional positive avalanche contribution (%)	11.4	11.1	11.1
Regional negative avalanche contribution (%)	-2.1	-2.2	-1.9
Median avalanche contribution (%)	3.7	4.7	4.8
20th percentile of avalanche contribution (%)	-2.7	-1.0	-0.3
80th percentile of avalanche contribution (%)	24.2	18.5	26.0

Supplementary Figures

Glacier-wide avalanche contribution and glacier variables

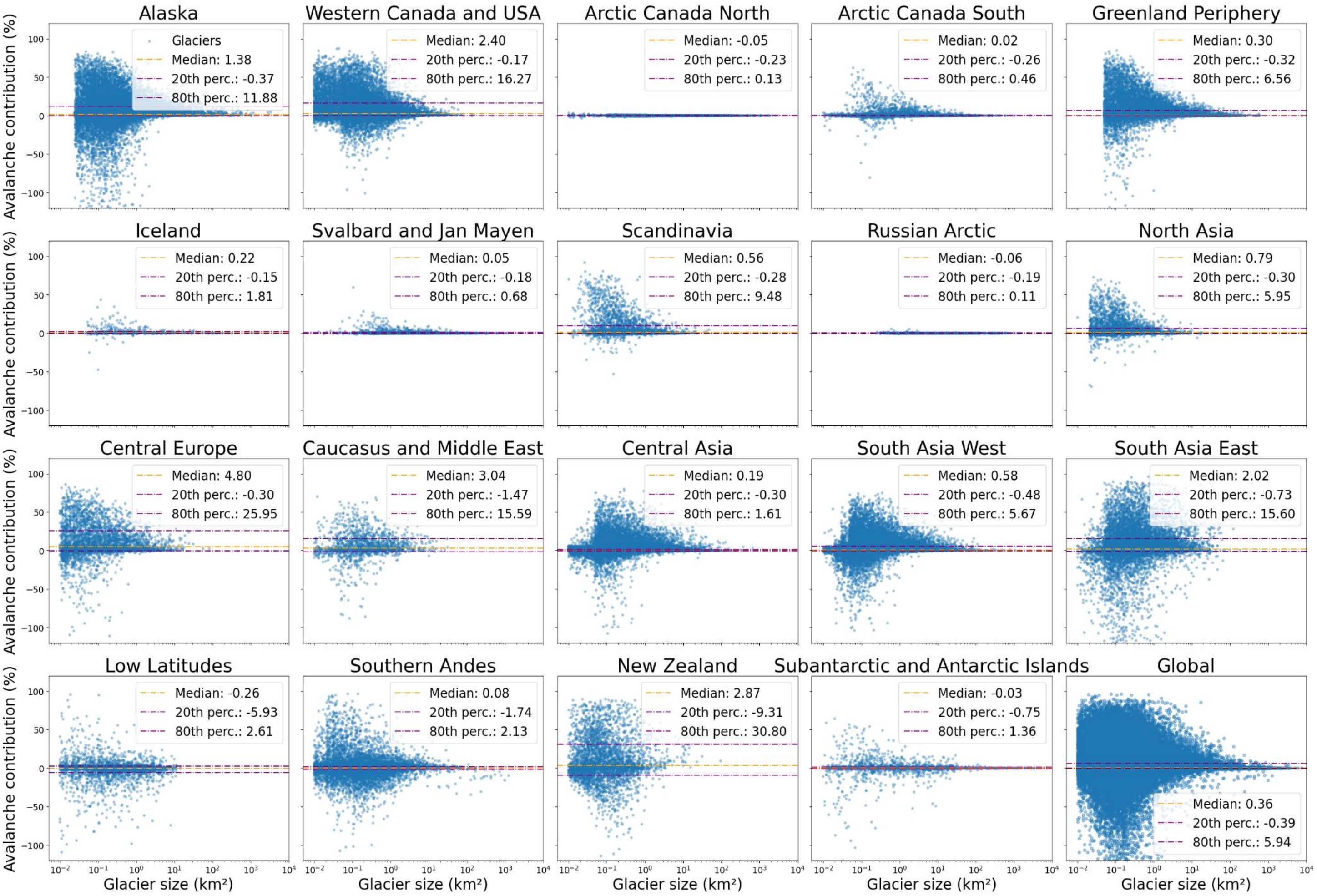

Supplementary Figure 1: Glacier-wide net avalanche contribution as a function of glacier size for each RGI region. *The yellow horizontal line corresponds to the median glacier-wide net avalanche contribution and the purple lines correspond to the 20th and 80th percentile values.*

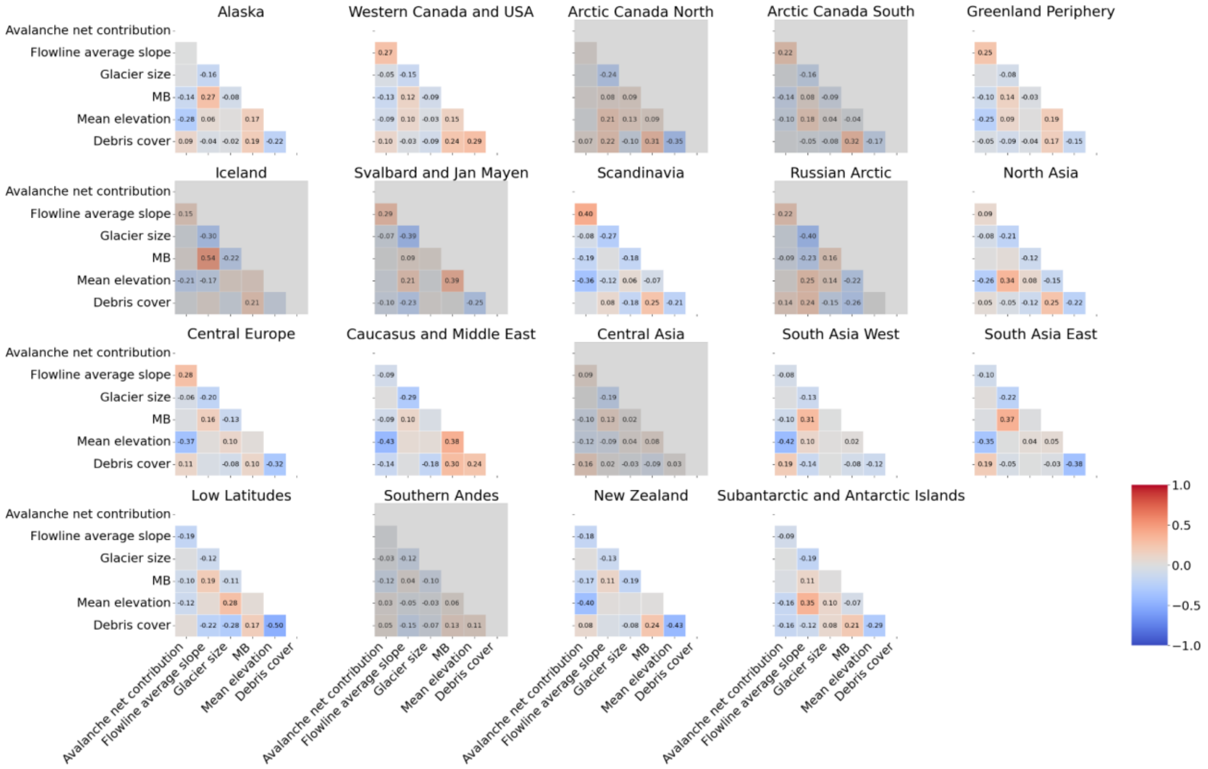

Supplementary Figure 2: Characteristics of avalanche-fed glaciers. *Correlation matrices of the glacier-wide net avalanche contribution, glacier average slope along the flowline, size, geodetic mass balance, mean elevation and percentage of debris cover for each RGI region. The values correspond to Pearson's correlation coefficients, and only those with a significant correlation (p-value < 0.01) are shown. Regions with a 20th and 80th percentile of the net avalanche contribution within -5% and +5%, which corresponds to a generally low influence from avalanches, are shaded in gray.*

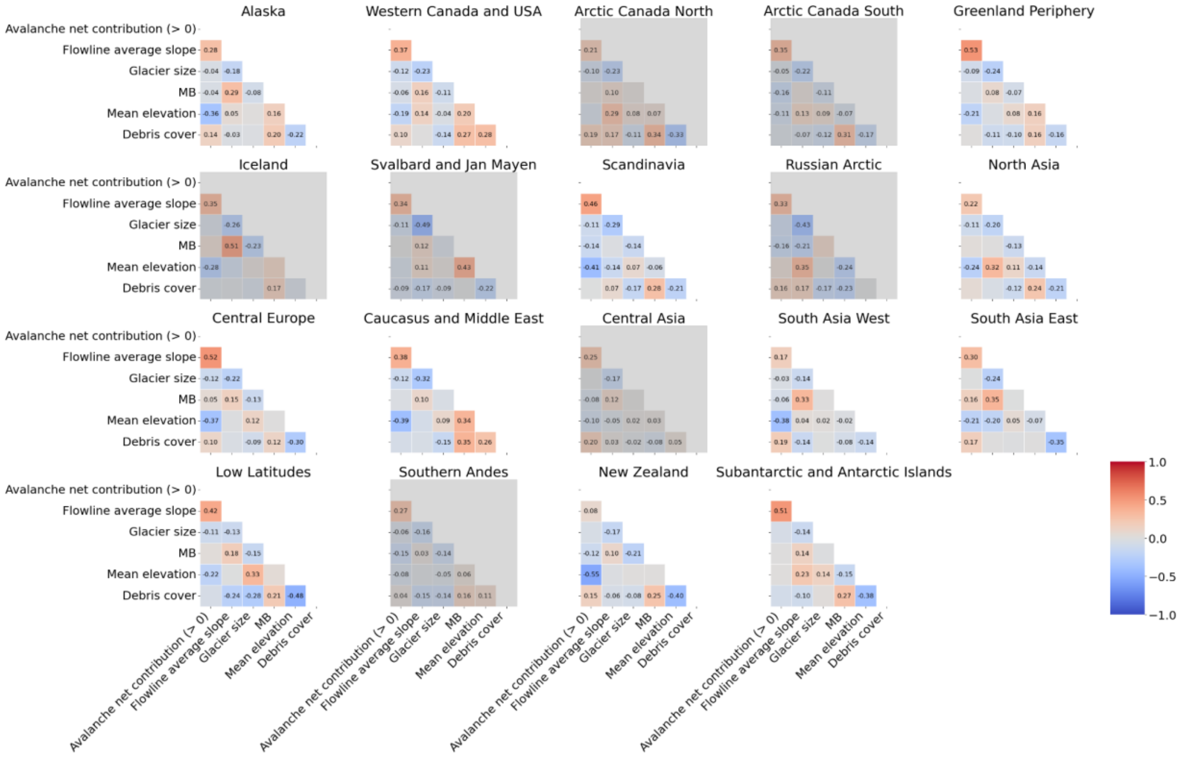

Supplementary Figure 3: Characteristics of positively avalanche-fed glaciers. *Same as Fig. S2 but only for a strictly positive glacier-wide net avalanche contribution to the glacier accumulation.*

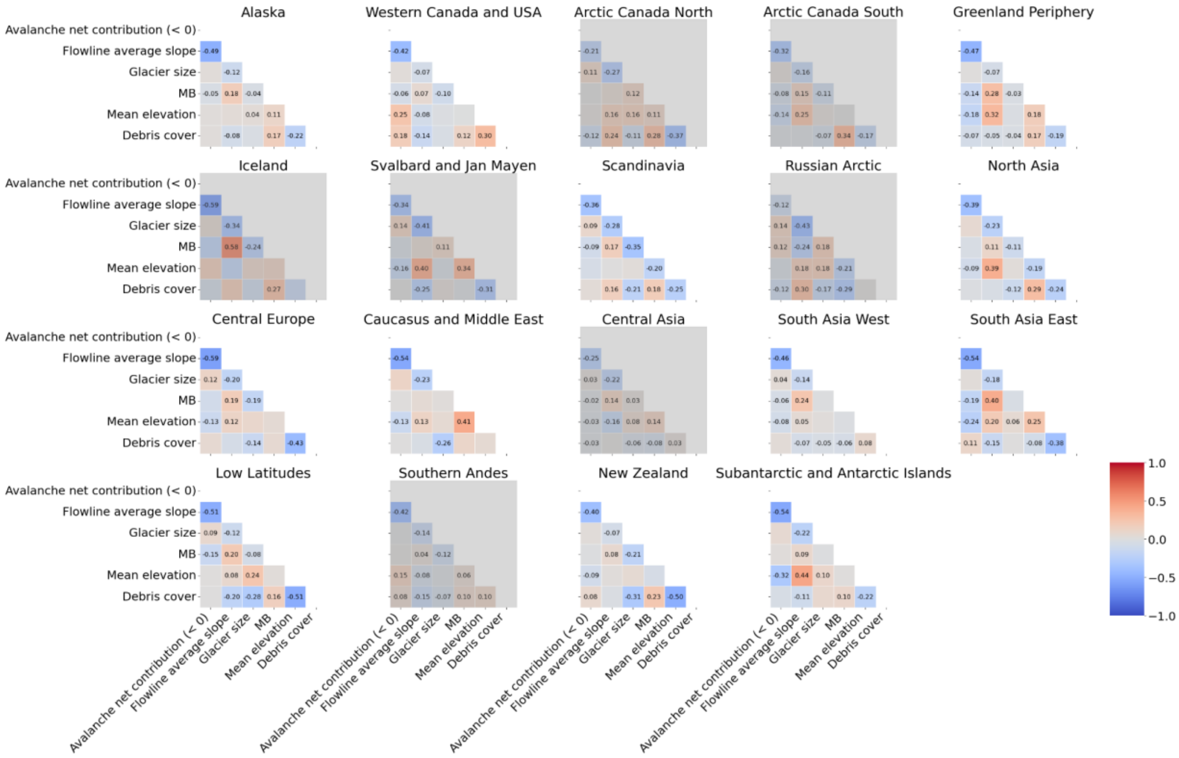

Supplementary Figure 4: Characteristics of negatively avalanche-fed glaciers. *Same as Fig. S2 but only for a strictly negative glacier-wide net avalanche contribution to the glacier accumulation.*

Influence of avalanches on the recalibration of the mass balance model

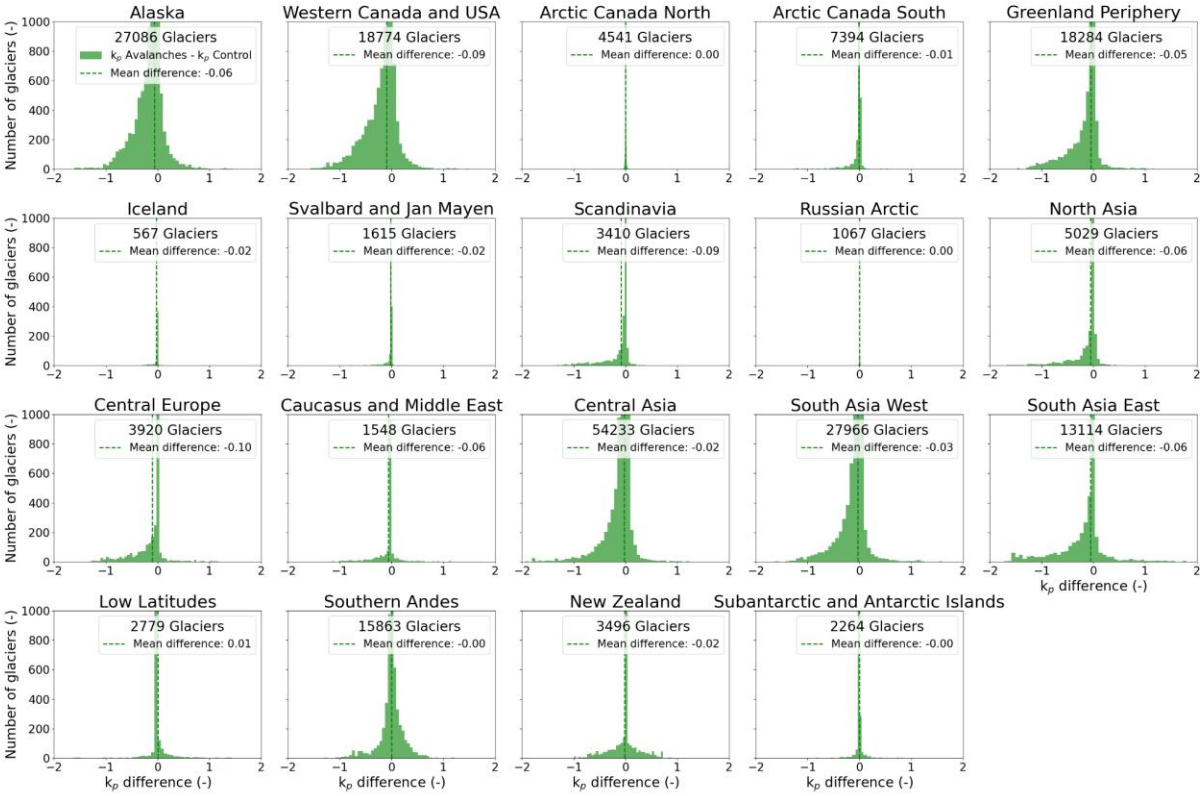

Supplementary Figure 5: Precipitation correction factor with and without avalanches. *Differences between precipitation correction factor (k_p) for all glaciers, with and without accounting for avalanches, after calibration of the 'Control' and 'Avalanches' models over the period 01/2000-12/2019. A negative value means that the precipitation correction factor is lower when explicitly accounting for avalanches.*

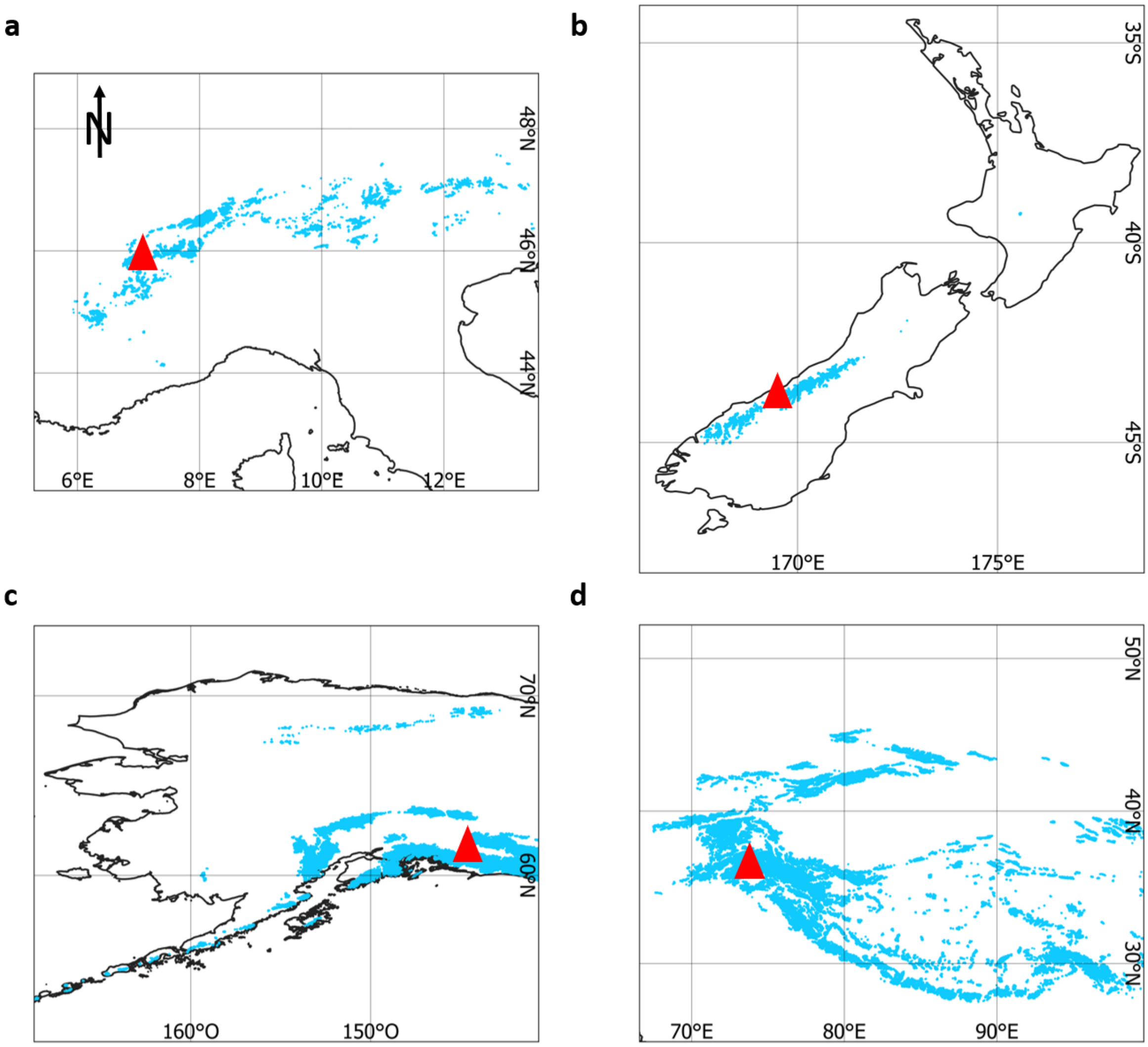

Supplementary Figure 6: Location of the four focus glaciers. *The glaciers are indicated by a red triangle and the blue outlines in the background show the RGI 6.0 glacier outlines in the corresponding regions:* ***a*** *Argentière Glacier (RGI6.0-11.03638; 45.951°N, 6.985°E),* ***b*** *Moore Glacier (RGI6.0-18.02066; -43.898°N, 169.786°E),* ***c*** *Black Glacier (RGI6.0-01.13640; 61.085°N, -143.831°E) and* ***d*** *Bazhin Glacier (RGI6.0-14.20029; 35.226°N, 74.637°E). RGI 6.0 glacier outlines (in blue) from [1], which is published under a CCBY license (https://creativecommons.org/licenses/by/4.0/), with no changes made. Land masks from OpenStreetMap (https://www.openstreetmap.org/copyright).*

Altitudinal mass balance profiles

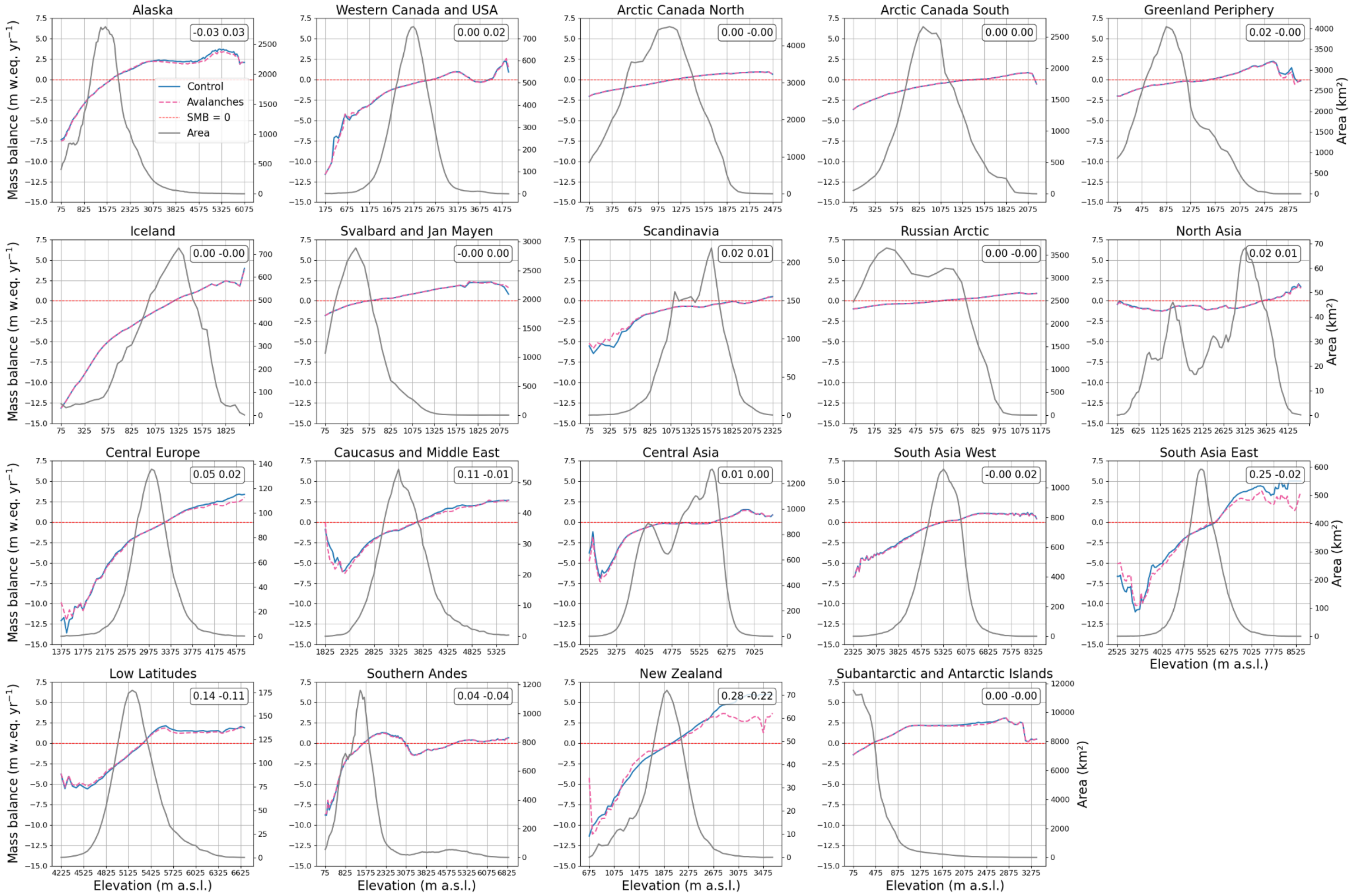

Supplementary Figure 7: Influence of avalanches on the regional mass balance altitudinal profiles. *Altitudinal mass balance profiles with (blue) and without (pink) avalanches for all RGI 6.0 regions. These profiles were obtained by taking the area-weighted average of the altitudinal profiles of all glaciers of the corresponding regions over the period 01/2000-12/2019. The gray line shows the glacier hypsometry and the red dashed line corresponds to a mass balance value of 0 m w.eq. yr^{-1}. The values in the upper right corner indicate the area-weighted mean difference between the mass balance with and without avalanches in the accumulation and the ablation zones.*

Projected ice volume changes

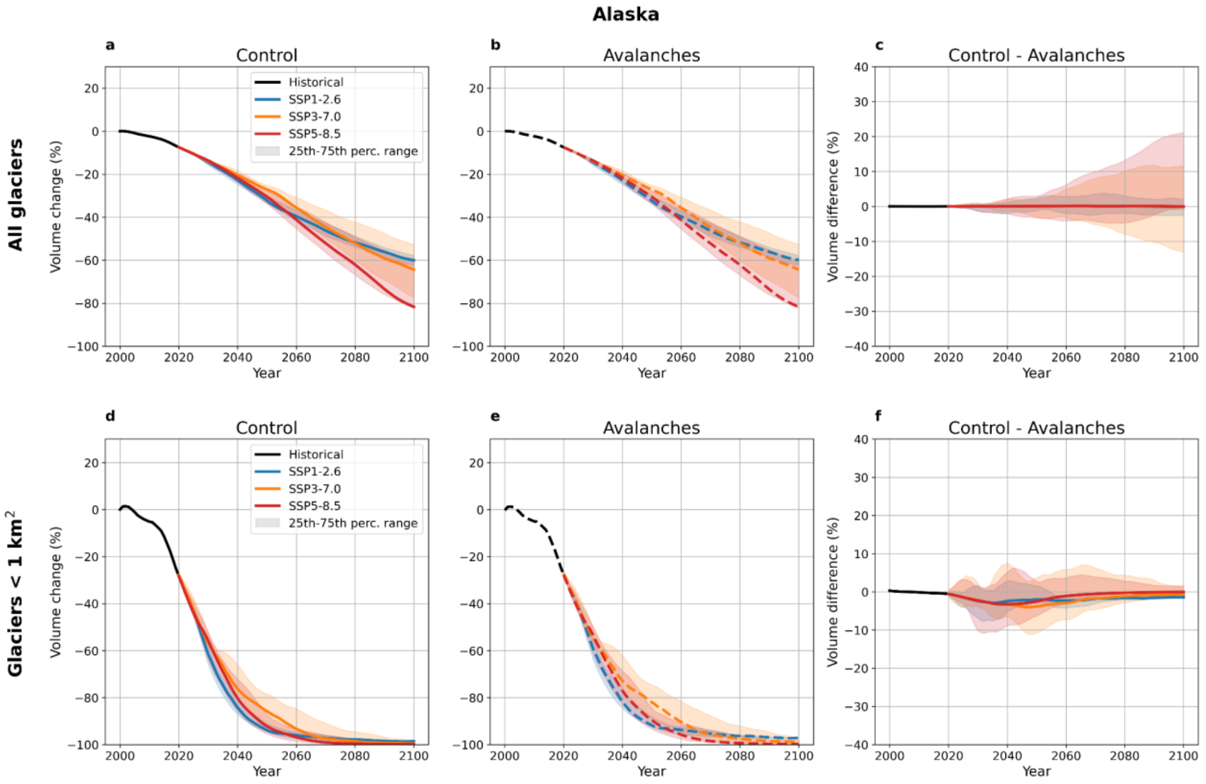

Supplementary Figure 8: Influence of avalanches on regional glacier volume changes in Alaska. *Projected ice volume changes of all glaciers (a-c) and the glaciers smaller than 1 km^2 (d-f) in Alaska, from the 'Control' (a, d) and 'Avalanches' (b, e) simulations. The right-hand plots (c, f) show the difference between the 'Control' and the 'Avalanches' simulations, so that negative values indicate more volume in the 'Avalanches' simulations. All percentages are given relative to the initial volume in 2000 in the 'Control' simulation. The black line corresponds to the historical period over which the mass balance model was calibrated using W5E5v2.0 data. The colored lines show the median future projections for different SSP scenarios, and the shaded areas indicate the 25th-75th percentile range. The different curves were smoothed using a 5-year rolling mean.*

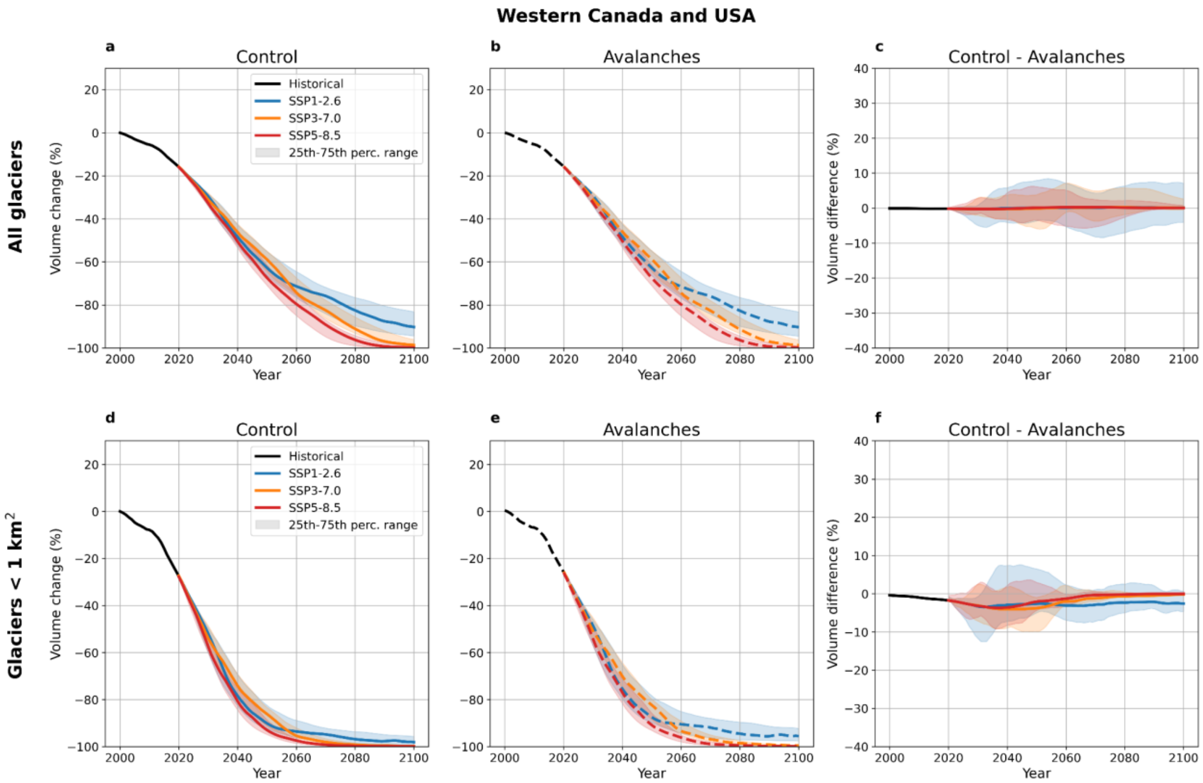

Supplementary Figure 9: Influence of avalanches on regional glacier volume changes in Western Canada and USA. *Projected ice volume changes of all glaciers (a-c) and the glaciers smaller than 1 km^2 (d-f) in Western Canada and USA, from the 'Control' (a, d) and 'Avalanches' (b, e) simulations. The right-hand plots (c, f) show the difference between the 'Control' and the 'Avalanches' simulations, so that negative values indicate more volume in the 'Avalanches' simulations. All percentages are given relative to the initial volume in 2000 in the 'Control' simulation. The black line corresponds to the historical period over which the mass balance model was calibrated using W5E5v2.0 data. The colored lines show the median future projections for different SSP scenarios, and the shaded areas indicate the 25th-75th percentile range. The different curves were smoothed using a 5-year rolling mean.*

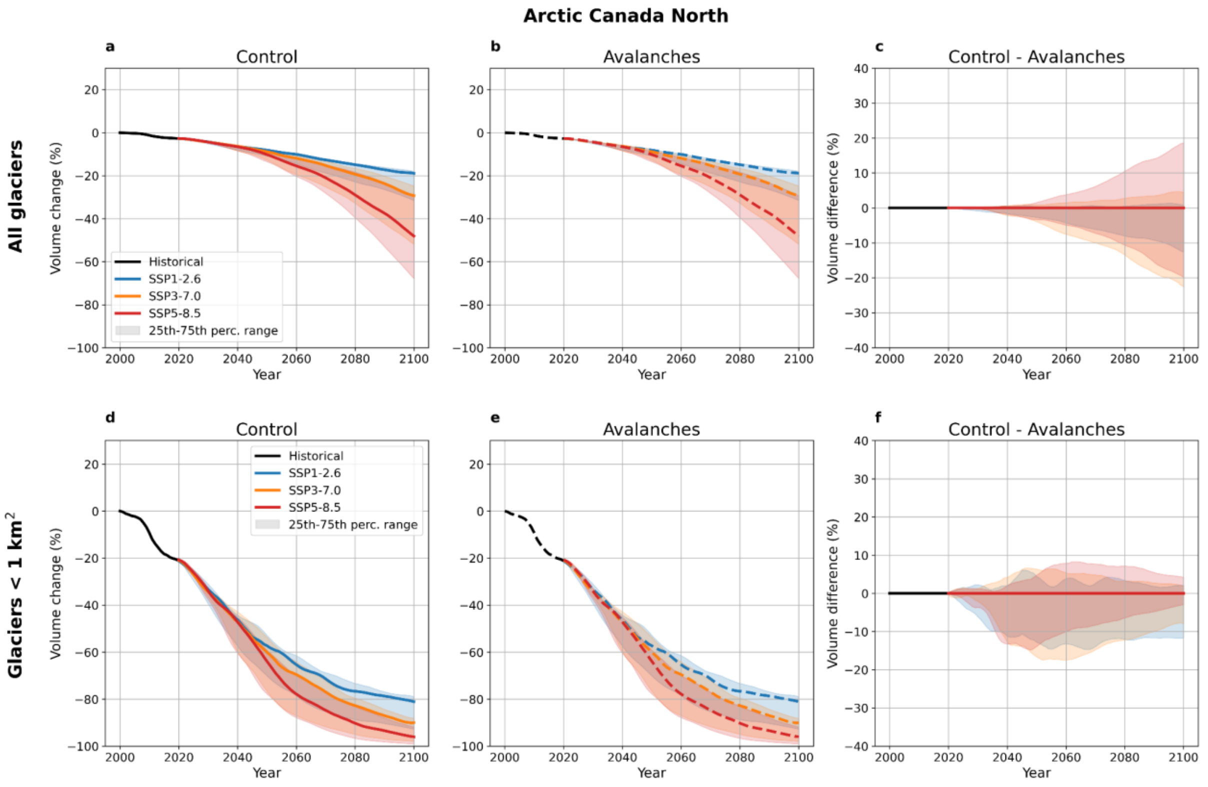

Supplementary Figure 10: Influence of avalanches on regional glacier volume changes in Arctic Canada North. *Projected ice volume changes of all glaciers (a-c) and the glaciers smaller than 1* km^2 *(d-f) in Arctic Canada North, from the 'Control' (a, d) and 'Avalanches' (b, e) simulations. The right-hand plots (c, f) show the difference between the 'Control' and the 'Avalanches' simulations, so that negative values indicate more volume in the 'Avalanches' simulations. All percentages are given relative to the initial volume in 2000 in the 'Control' simulation. The black line corresponds to the historical period over which the mass balance model was calibrated using W5E5v2.0 data. The colored lines show the median future projections for different SSP scenarios, and the shaded areas indicate the 25th-75th percentile range. The different curves were smoothed using a 5-year rolling mean.*

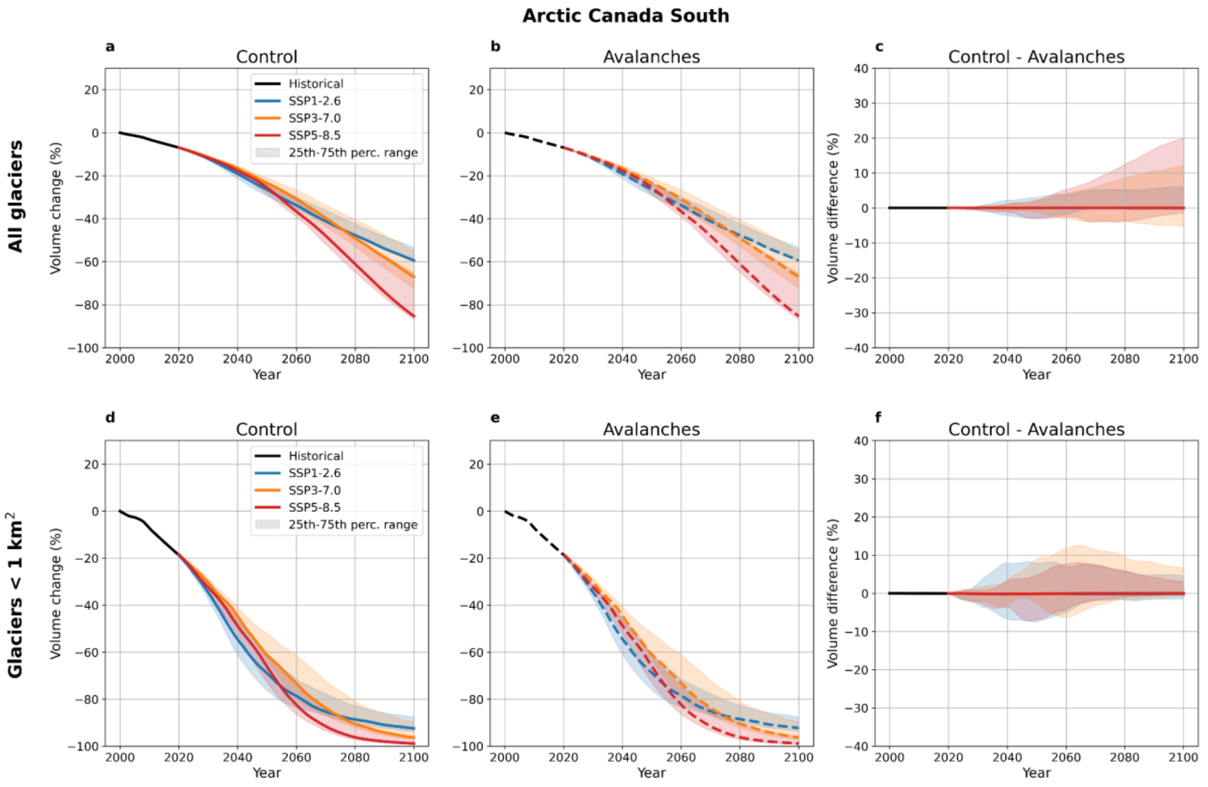

Supplementary Figure 11: Influence of avalanches on regional glacier volume changes in Arctic Canada South. *Projected ice volume changes of all glaciers (a-c) and the glaciers smaller than 1 km^2 (d-f) in Arctic Canada South, from the 'Control' (a, d) and 'Avalanches' (b, e) simulations. The right-hand plots (c, f) show the difference between the 'Control' and the 'Avalanches' simulations, so that negative values indicate more volume in the 'Avalanches' simulations. All percentages are given relative to the initial volume in 2000 in the 'Control' simulation. The black line corresponds to the historical period over which the mass balance model was calibrated using W5E5v2.0 data. The colored lines show the median future projections for different SSP scenarios, and the shaded areas indicate the 25th-75th percentile range. The different curves were smoothed using a 5-year rolling mean.*

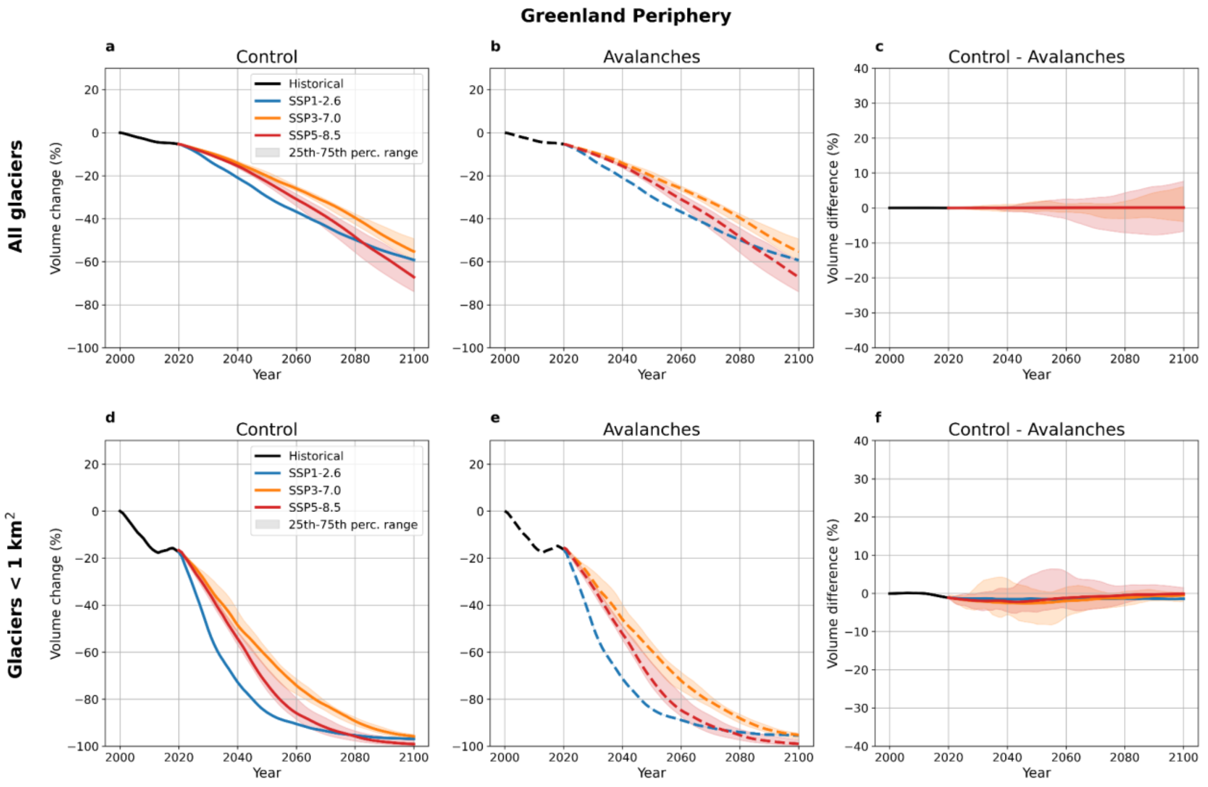

Supplementary Figure 12: Influence of avalanches on regional glacier volume changes in the Greenland Periphery. *Projected ice volume changes of all glaciers (a-c) and the glaciers smaller than 1 km^2 (d-f) in the Greenland Periphery, from the 'Control' (a, d) and 'Avalanches' (b, e) simulations. The right-hand plots (c, f) show the difference between the 'Control' and the 'Avalanches' simulations, so that negative values indicate more volume in the 'Avalanches' simulations. All percentages are given relative to the initial volume in 2000 in the 'Control' simulation. The black line corresponds to the historical period over which the mass balance model was calibrated using W5E5v2.0 data. The colored lines show the median future projections for different SSP scenarios, and the shaded areas indicate the 25th-75th percentile range. The different curves were smoothed using a 5-year rolling mean.*

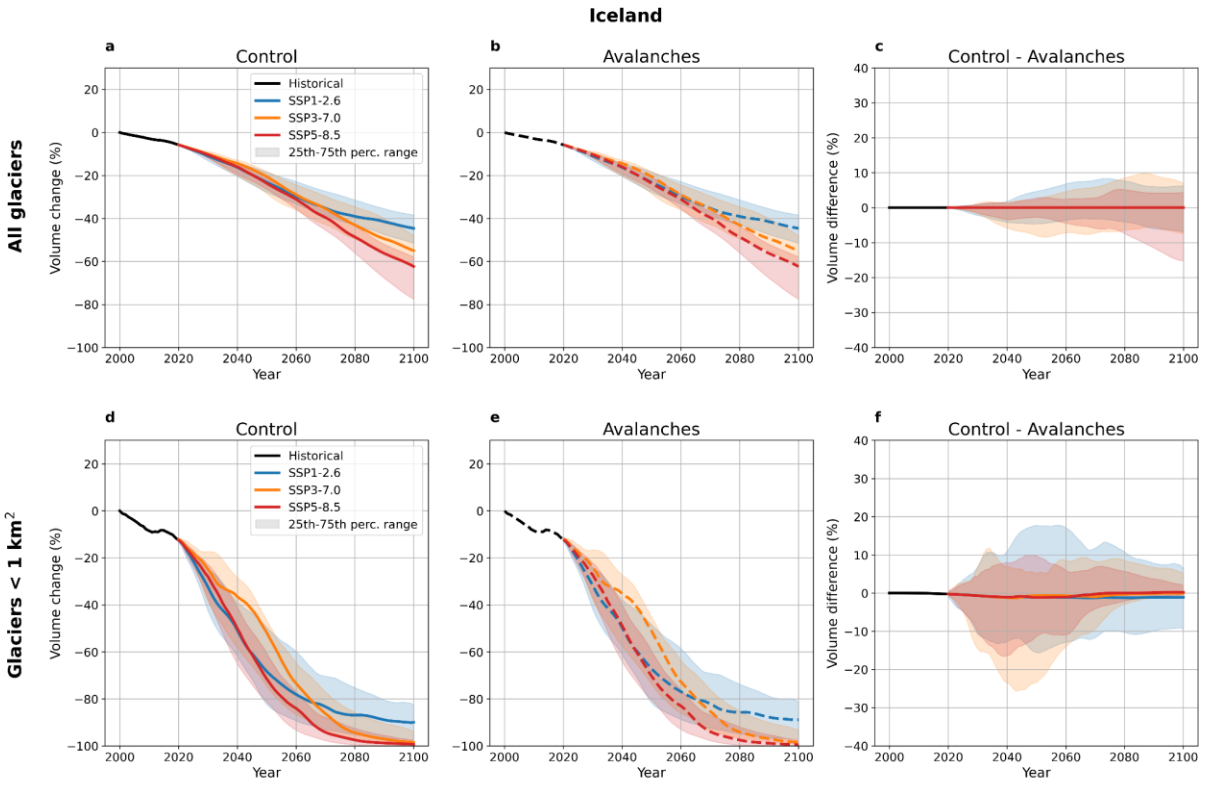

Supplementary Figure 13: Influence of avalanches on regional glacier volume changes in Iceland. *Projected ice volume changes of all glaciers (a-c) and the glaciers smaller than 1 km^2 (d-f) in Iceland, from the 'Control' (a, d) and 'Avalanches' (b, e) simulations. The right-hand plots (c, f) show the difference between the 'Control' and the 'Avalanches' simulations, so that negative values indicate more volume in the 'Avalanches' simulations. All percentages are given relative to the initial volume in 2000 in the 'Control' simulation. The black line corresponds to the historical period over which the mass balance model was calibrated using W5E5v2.0 data. The colored lines show the median future projections for different SSP scenarios, and the shaded areas indicate the 25th-75th percentile range. The different curves were smoothed using a 5-year rolling mean.*

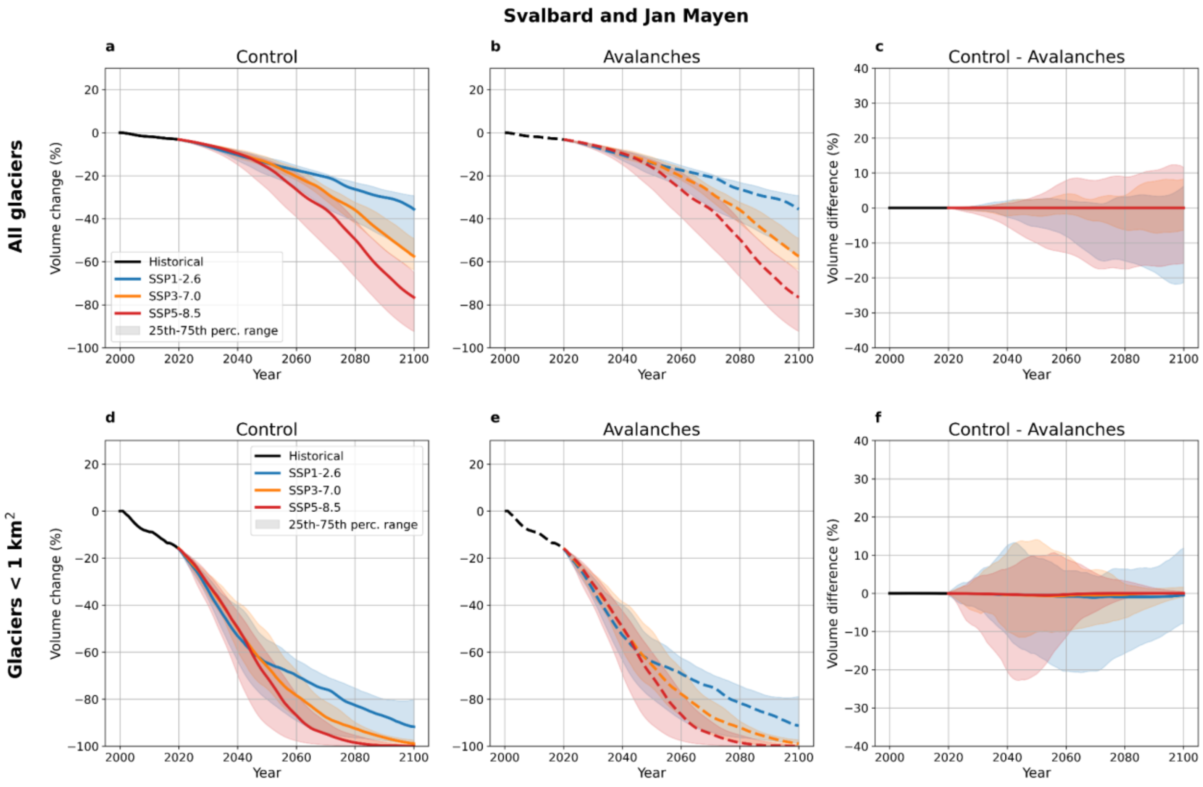

Supplementary Figure 14: Influence of avalanches on regional glacier volume changes in Svalbard and Jan Mayen. *Projected ice volume changes of all glaciers (a-c) and the glaciers smaller than 1 km^2 (d-f) in Svalbard and Jan Mayen, from the 'Control' (a, d) and 'Avalanches' (b, e) simulations. The right-hand plots (c, f) show the difference between the 'Control' and the 'Avalanches' simulations, so that negative values indicate more volume in the 'Avalanches' simulations. All percentages are given relative to the initial volume in 2000 in the 'Control' simulation. The black line corresponds to the historical period over which the mass balance model was calibrated using W5E5v2.0 data. The colored lines show the median future projections for different SSP scenarios, and the shaded areas indicate the 25th-75th percentile range. The different curves were smoothed using a 5-year rolling mean.*

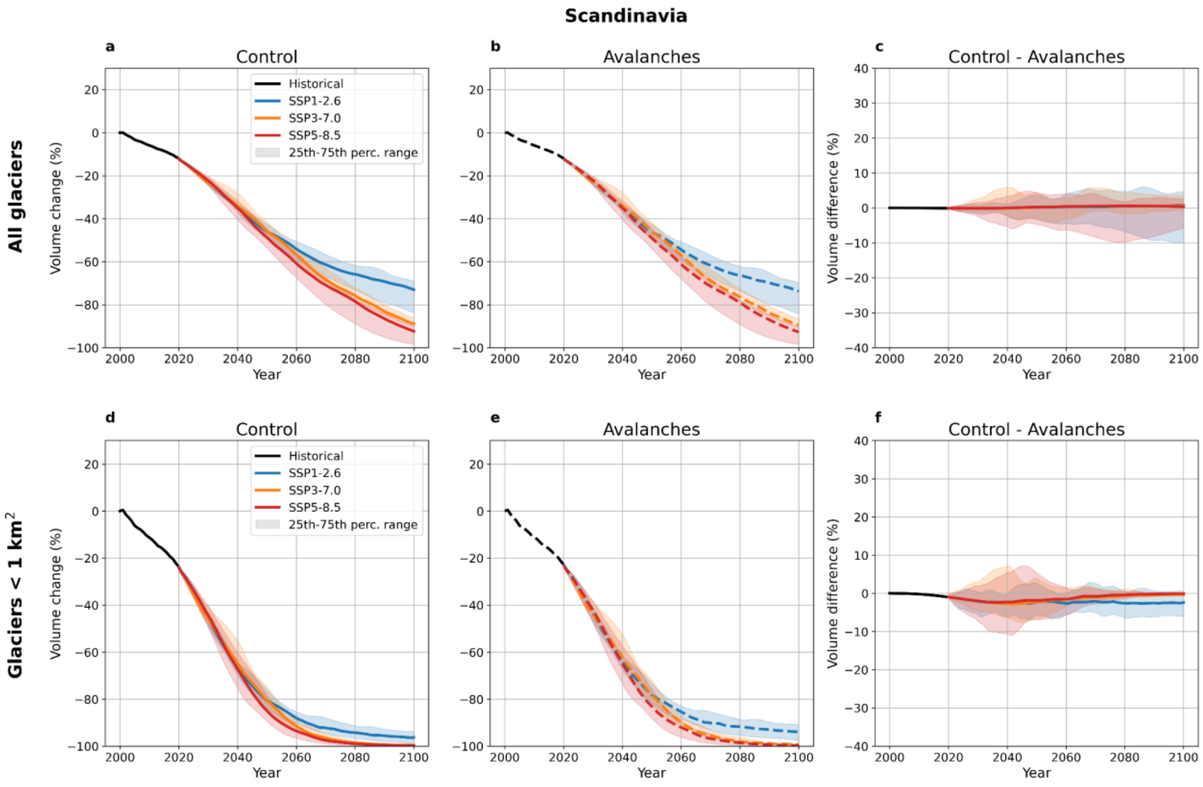

Supplementary Figure 15: Influence of avalanches on regional glacier volume changes in Scandinavia. *Projected ice volume changes of all glaciers (a-c) and the glaciers smaller than 1 km^2 (d-f) in Scandinavia, from the 'Control' (a, d) and 'Avalanches' (b, e) simulations. The right-hand plots (c, f) show the difference between the 'Control' and the 'Avalanches' simulations, so that negative values indicate more volume in the 'Avalanches' simulations. All percentages are given relative to the initial volume in 2000 in the 'Control' simulation. The black line corresponds to the historical period over which the mass balance model was calibrated using W5E5v2.0 data. The colored lines show the median future projections for different SSP scenarios, and the shaded areas indicate the 25th-75th percentile range. The different curves were smoothed using a 5-year rolling mean.*

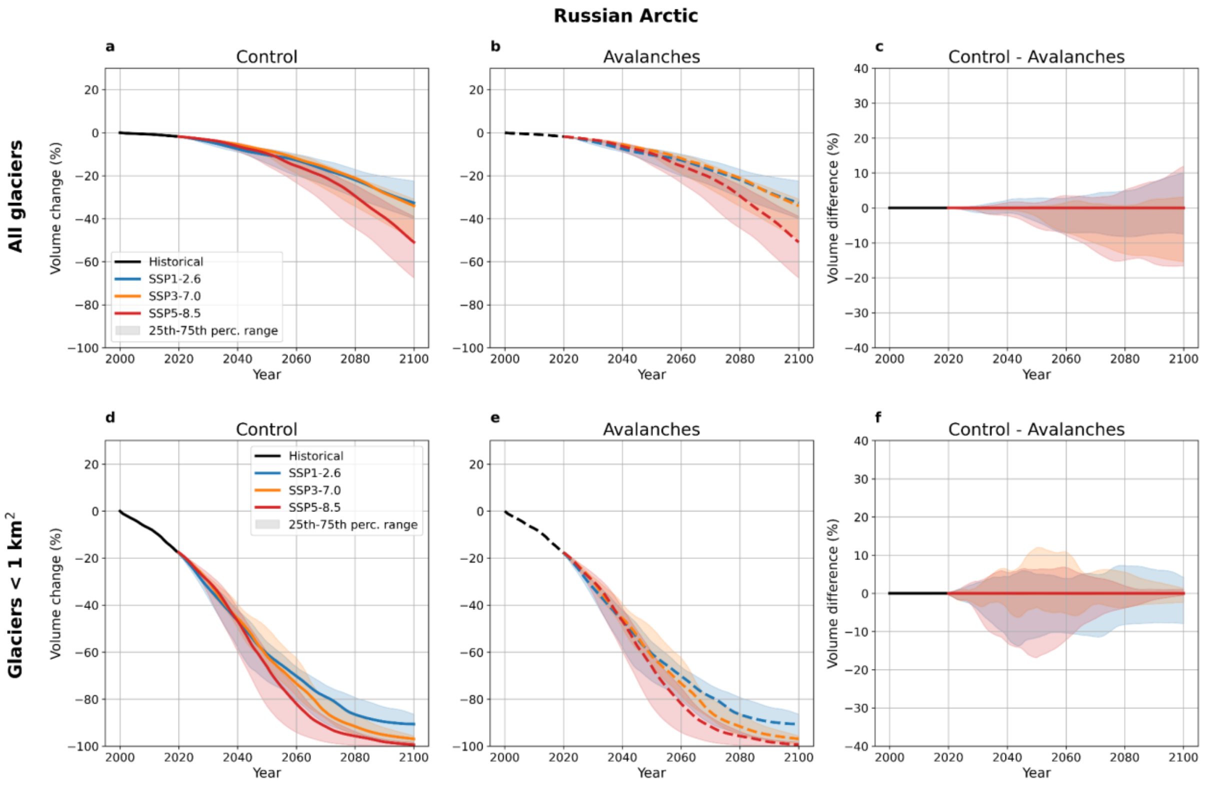

Supplementary Figure 16: Influence of avalanches on regional glacier volume changes in the Russian Arctic. *Projected ice volume changes of all glaciers (a-c) and the glaciers smaller than 1* km^2 *(d-f) in the Russian Arctic, from the 'Control' (a, d) and 'Avalanches' (b, e) simulations. The right-hand plots (c, f) show the difference between the 'Control' and the 'Avalanches' simulations, so that negative values indicate more volume in the 'Avalanches' simulations. All percentages are given relative to the initial volume in 2000 in the 'Control' simulation. The black line corresponds to the historical period over which the mass balance model was calibrated using W5E5v2.0 data. The colored lines show the median future projections for different SSP scenarios, and the shaded areas indicate the 25th-75th percentile range. The different curves were smoothed using a 5-year rolling mean.*

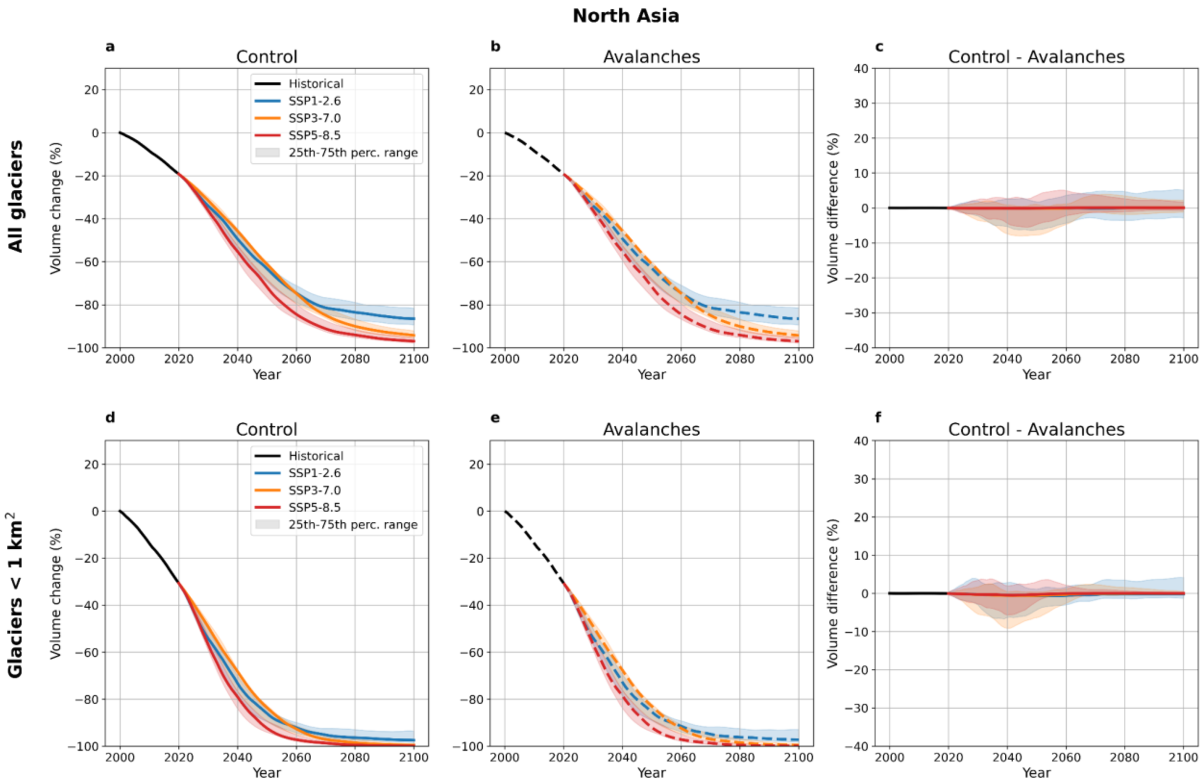

Supplementary Figure 17: Influence of avalanches on regional glacier volume changes in North Asia. *Projected ice volume changes of all glaciers (a-c) and the glaciers smaller than 1 km^2 (d-f) in North Asia, from the 'Control' (a, d) and 'Avalanches' (b, e) simulations. The right-hand plots (c, f) show the difference between the 'Control' and the 'Avalanches' simulations, so that negative values indicate more volume in the 'Avalanches' simulations. All percentages are given relative to the initial volume in 2000 in the 'Control' simulation. The black line corresponds to the historical period over which the mass balance model was calibrated using W5E5v2.0 data. The colored lines show the median future projections for different SSP scenarios, and the shaded areas indicate the 25th-75th percentile range. The different curves were smoothed using a 5-year rolling mean.*

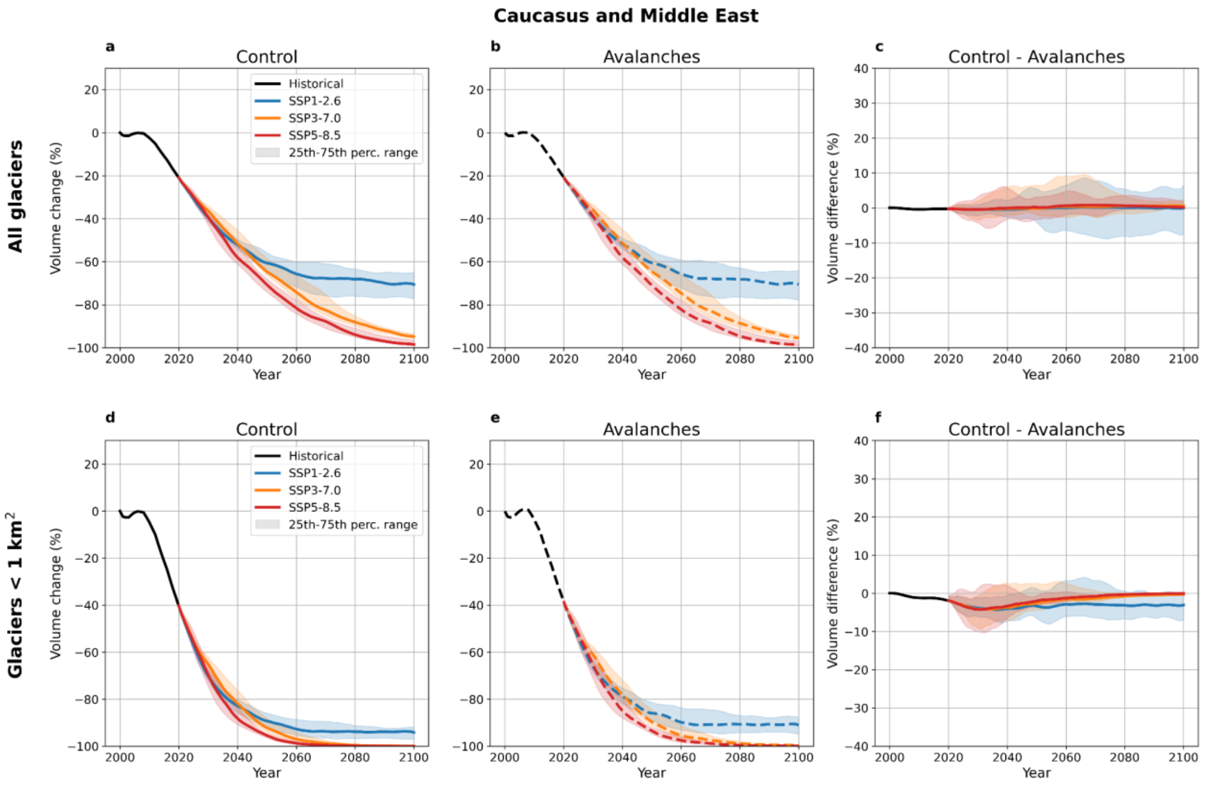

Supplementary Figure 18: Influence of avalanches on regional glacier volume changes in Caucasus and the Middle East. *Projected ice volume changes of all glaciers (a-c) and the glaciers smaller than 1 km^2 (d-f) in Caucasus and the Middle East, from the 'Control' (a, d) and 'Avalanches' (b, e) simulations. The right-hand plots (c, f) show the difference between the 'Control' and the 'Avalanches' simulations, so that negative values indicate more volume in the 'Avalanches' simulations. All percentages are given relative to the initial volume in 2000 in the 'Control' simulation. The black line corresponds to the historical period over which the mass balance model was calibrated using W5E5v2.0 data. The colored lines show the median future projections for different SSP scenarios, and the shaded areas indicate the 25th-75th percentile range. The different curves were smoothed using a 5-year rolling mean.*

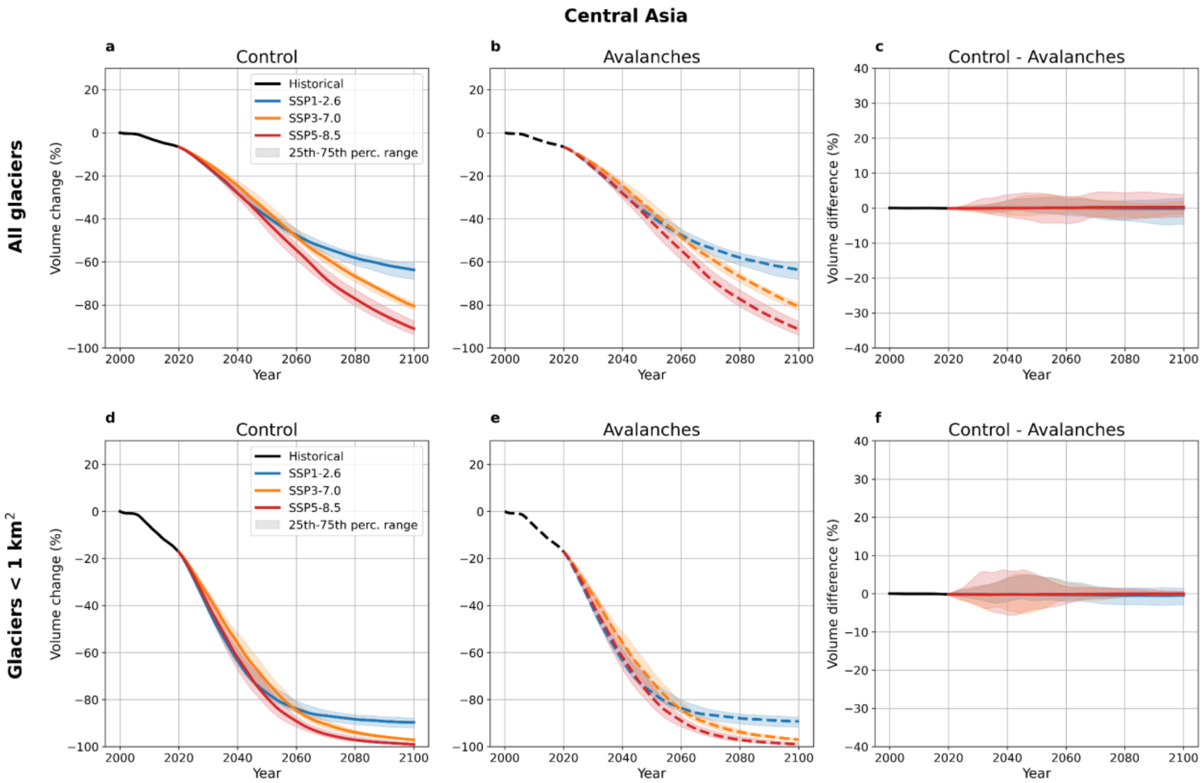

Supplementary Figure 19: Influence of avalanches on regional glacier volume changes in Central Asia. *Projected ice volume changes of all glaciers (a-c) and the glaciers smaller than 1* km^2 *(d-f) in Central Asia, from the 'Control' (a, d) and 'Avalanches' (b, e) simulations. The right-hand plots (c, f) show the difference between the 'Control' and the 'Avalanches' simulations, so that negative values indicate more volume in the 'Avalanches' simulations. All percentages are given relative to the initial volume in 2000 in the 'Control' simulation. The black line corresponds to the historical period over which the mass balance model was calibrated using W5E5v2.0 data. The colored lines show the median future projections for different SSP scenarios, and the shaded areas indicate the 25th-75th percentile range. The different curves were smoothed using a 5-year rolling mean.*

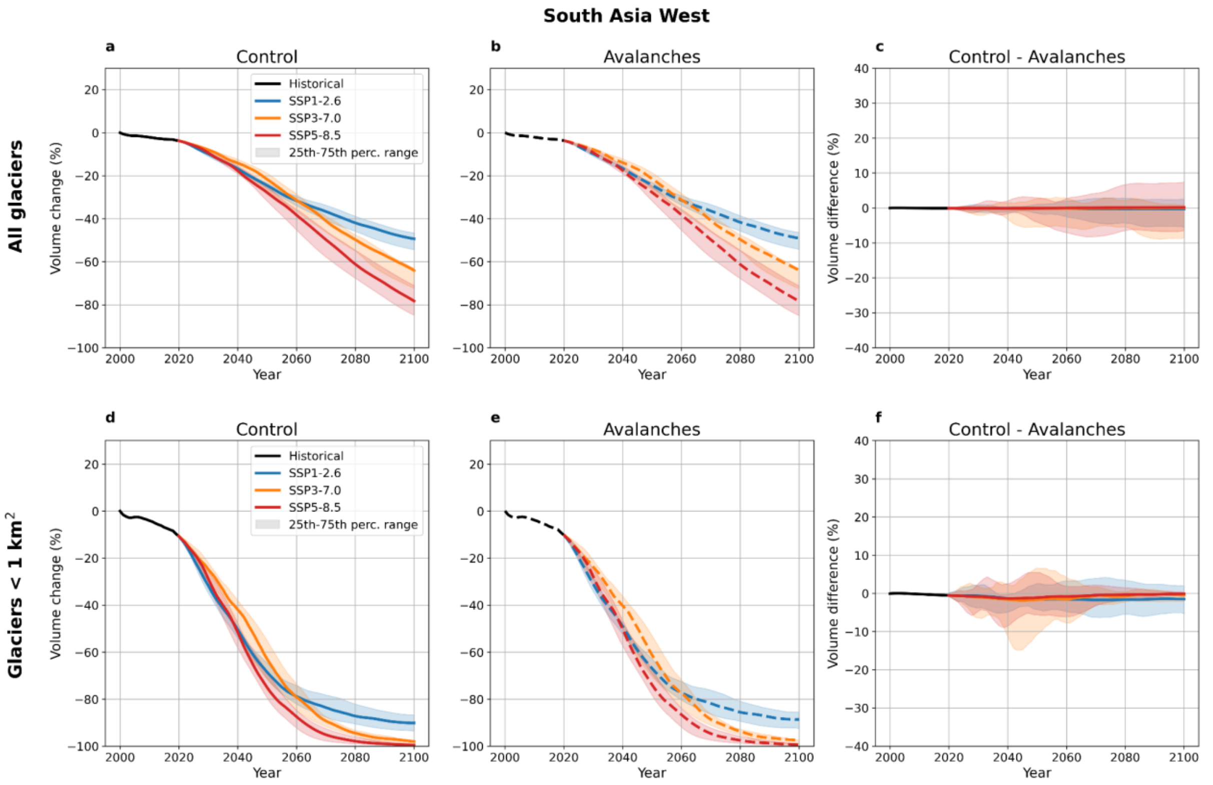

Supplementary Figure 20: Influence of avalanches on regional glacier volume changes in South Asia West. *Projected ice volume changes of all glaciers (a-c) and the glaciers smaller than 1 km^2 (d-f) in South Asia West, from the 'Control' (a, d) and 'Avalanches' (b, e) simulations. The right-hand plots (c, f) show the difference between the 'Control' and the 'Avalanches' simulations, so that negative values indicate more volume in the 'Avalanches' simulations. All percentages are given relative to the initial volume in 2000 in the 'Control' simulation. The black line corresponds to the historical period over which the mass balance model was calibrated using W5E5v2.0 data. The colored lines show the median future projections for different SSP scenarios, and the shaded areas indicate the 25th-75th percentile range. The different curves were smoothed using a 5-year rolling mean.*

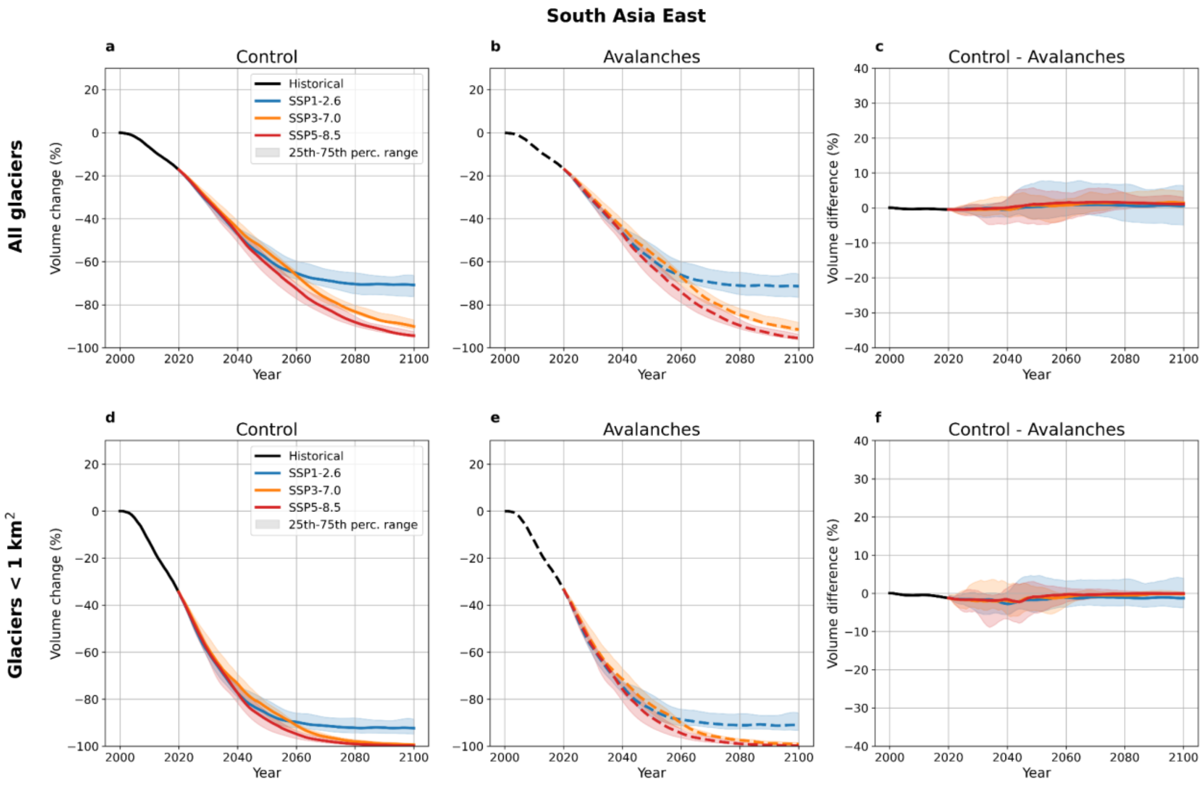

Supplementary Figure 21: Influence of avalanches on regional glacier volume changes in South Asia East. *Projected ice volume changes of all glaciers (a-c) and the glaciers smaller than 1 km^2 (d-f) in South Asia East, from the 'Control' (a, d) and 'Avalanches' (b, e) simulations. The right-hand plots (c, f) show the difference between the 'Control' and the 'Avalanches' simulations, so that negative values indicate more volume in the 'Avalanches' simulations. All percentages are given relative to the initial volume in 2000 in the 'Control' simulation. The black line corresponds to the historical period over which the mass balance model was calibrated using W5E5v2.0 data. The colored lines show the median future projections for different SSP scenarios, and the shaded areas indicate the 25th-75th percentile range. The different curves were smoothed using a 5-year rolling mean.*

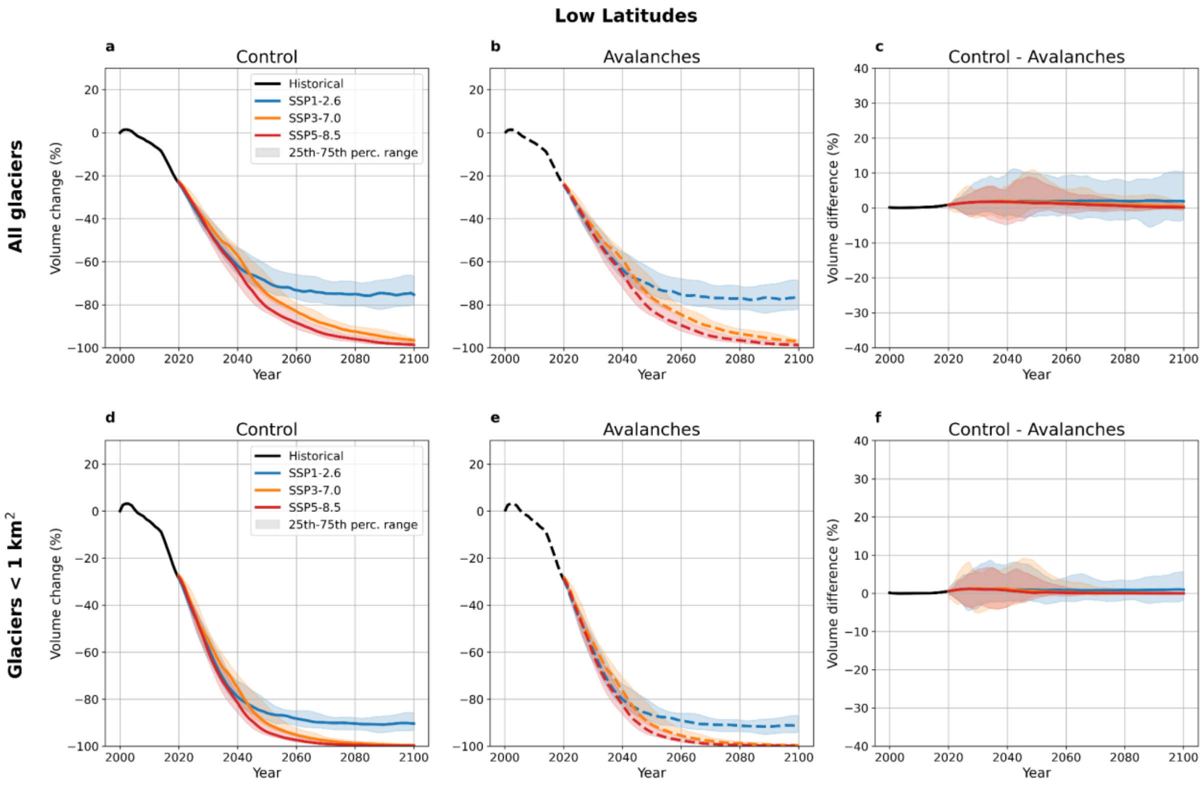

Supplementary Figure 22: Influence of avalanches on regional glacier volume changes in the Low Latitudes. *Projected ice volume changes of all glaciers (a-c) and the glaciers smaller than 1 km^2 (d-f) in the Low Latitudes, from the 'Control' (a, d) and 'Avalanches' (b, e) simulations. The right-hand plots (c, f) show the difference between the 'Control' and the 'Avalanches' simulations, so that negative values indicate more volume in the 'Avalanches' simulations. All percentages are given relative to the initial volume in 2000 in the 'Control' simulation. The black line corresponds to the historical period over which the mass balance model was calibrated using W5E5v2.0 data. The colored lines show the median future projections for different SSP scenarios, and the shaded areas indicate the 25th-75th percentile range. The different curves were smoothed using a 5-year rolling mean.*

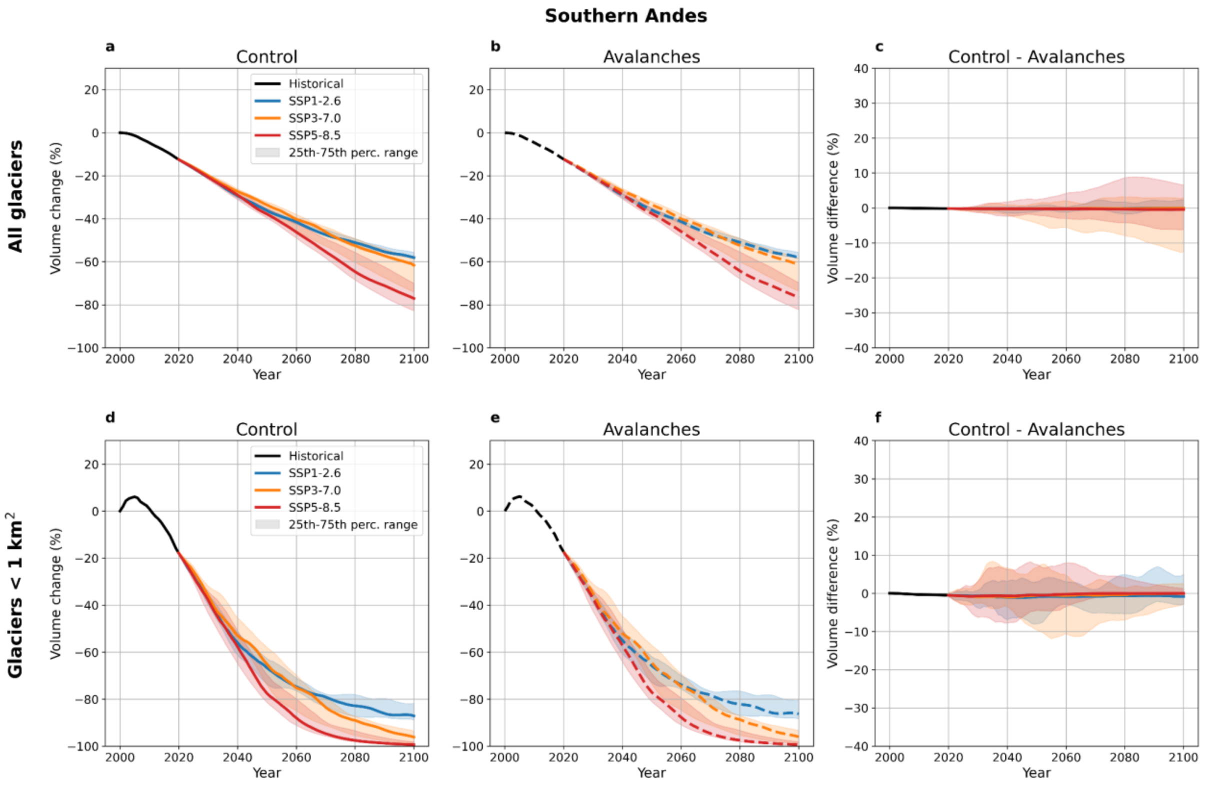

Supplementary Figure 23: Influence of avalanches on regional glacier volume changes in the Southern Andes. *Projected ice volume changes of all glaciers (a-c) and the glaciers smaller than 1 km^2 (d-f) in the Southern Andes, from the 'Control' (a, d) and 'Avalanches' (b, e) simulations. The right-hand plots (c, f) show the difference between the 'Control' and the 'Avalanches' simulations, so that negative values indicate more volume in the 'Avalanches' simulations. All percentages are given relative to the initial volume in 2000 in the 'Control' simulation. The black line corresponds to the historical period over which the mass balance model was calibrated using W5E5v2.0 data. The colored lines show the median future projections for different SSP scenarios, and the shaded areas indicate the 25th-75th percentile range. The different curves were smoothed using a 5-year rolling mean.*

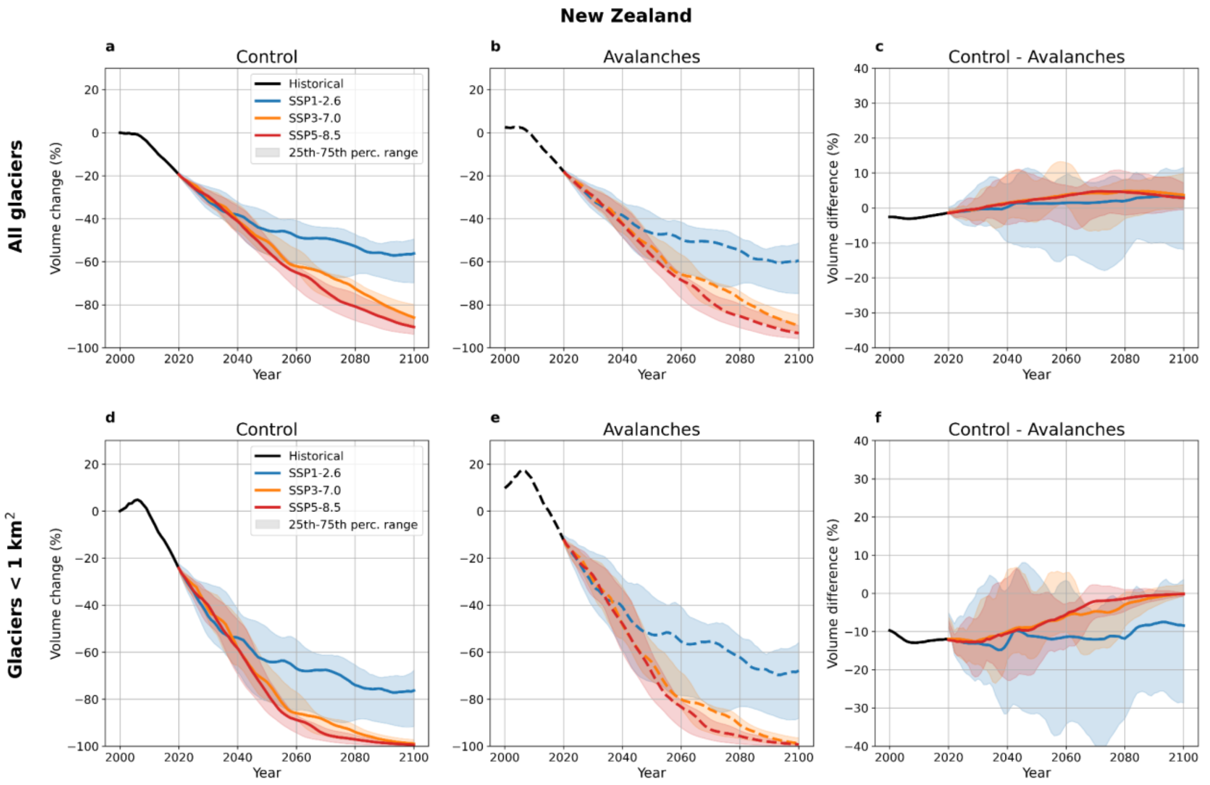

Supplementary Figure 24: Influence of avalanches on regional glacier volume changes in New Zealand. *Projected ice volume changes of all glaciers (a-c) and the glaciers smaller than 1 km^2 (d-f) in New Zealand, from the 'Control' (a, d) and 'Avalanches' (b, e) simulations. The right-hand plots (c, f) show the difference between the 'Control' and the 'Avalanches' simulations, so that negative values indicate more volume in the 'Avalanches' simulations. All percentages are given relative to the initial volume in 2000 in the 'Control' simulation. The black line corresponds to the historical period over which the mass balance model was calibrated using W5E5v2.0 data. The colored lines show the median future projections for different SSP scenarios, and the shaded areas indicate the 25th-75th percentile range. The different curves were smoothed using a 5-year rolling mean.*

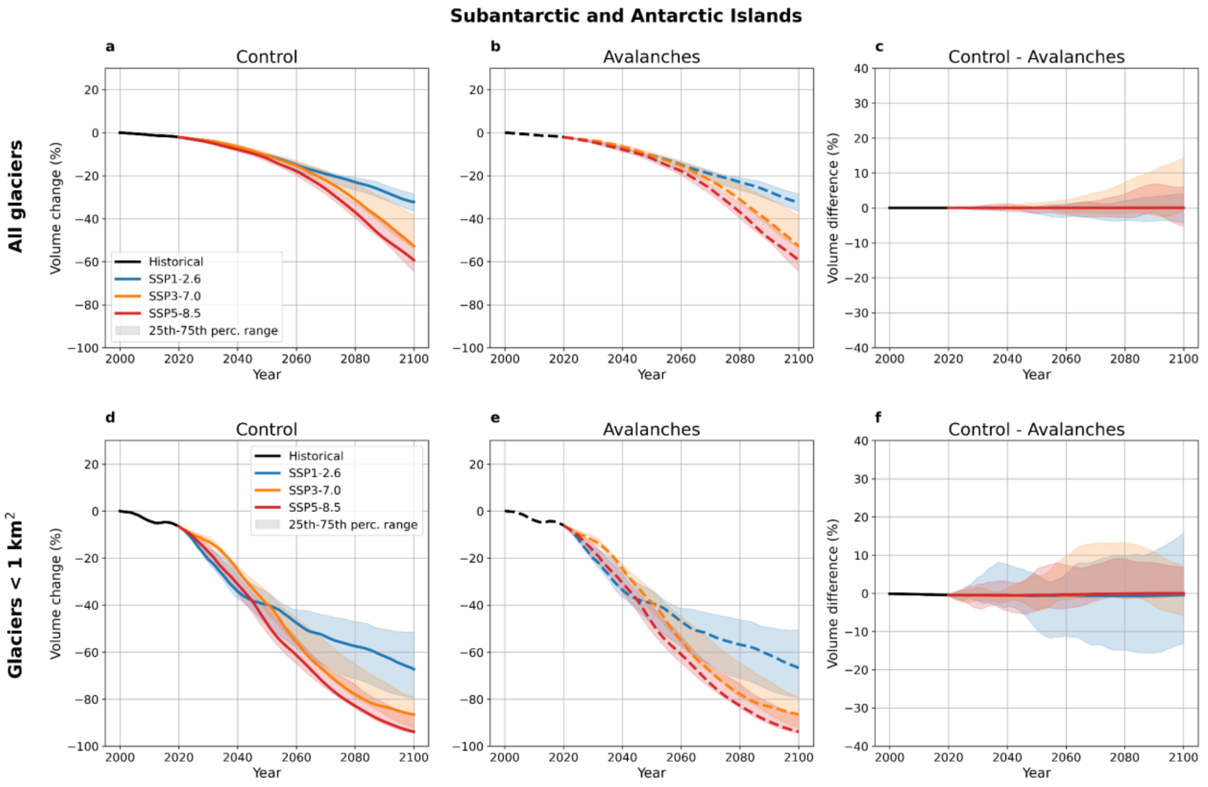

Supplementary Figure 25: Influence of avalanches on regional glacier volume changes in the Subantarctic and Antarctic Islands. *Projected ice volume changes of all glaciers (a-c) and the glaciers smaller than 1 km^2 (d-f) in the Subantarctic and Antarctic Islands, from the 'Control' (a, d) and 'Avalanches' (b, e) simulations. The right-hand plots (c, f) show the difference between the 'Control' and the 'Avalanches' simulations, so that negative values indicate more volume in the 'Avalanches' simulations. All percentages are given relative to the initial volume in 2000 in the 'Control' simulation. The black line corresponds to the historical period over which the mass balance model was calibrated using W5E5v2.0 data. The colored lines show the median future projections for different SSP scenarios, and the shaded areas indicate the 25th-75th percentile range. The different curves were smoothed using a 5-year rolling mean.*

Projected avalanche contribution changes

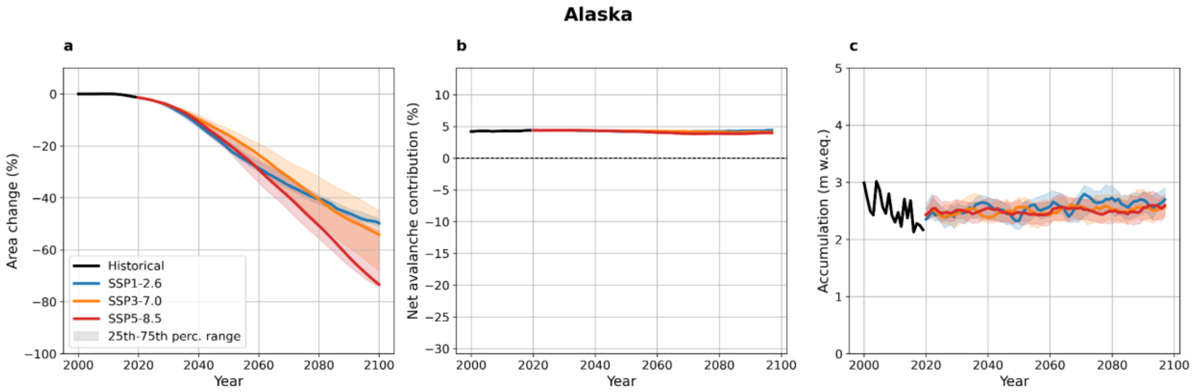

Supplementary Figure 26: Future evolution of regional avalanche contribution in Alaska. *Projected changes of glacier area (a), avalanche contribution to accumulation (b) and total snow accumulation (c) for Alaska. The black line corresponds to the historical period over which the mass balance model was calibrated using W5E5v2.0 data. The colored lines show the median future projections for different SSP scenarios, and the shaded areas indicate the 25th-75th percentile range. The different curves were smoothed using a 5-year rolling mean, except for the historical accumulation.*

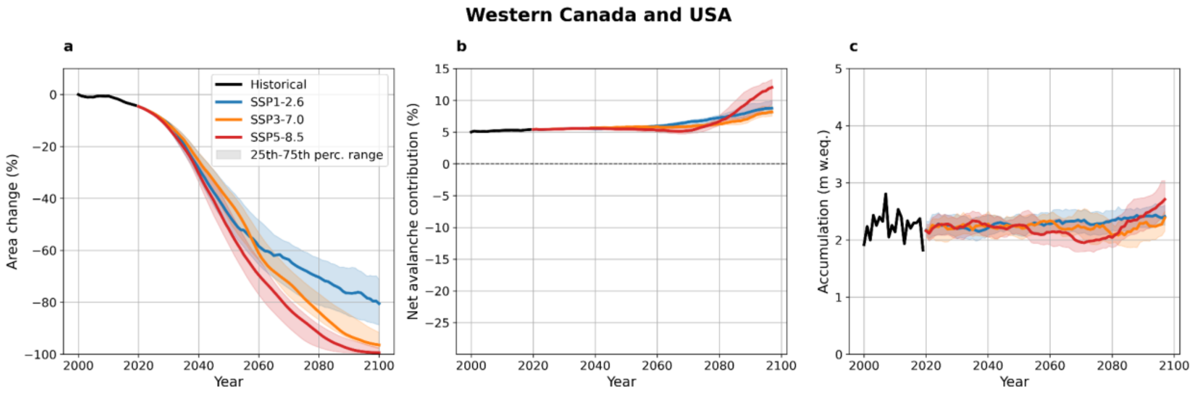

Supplementary Figure 27: Future evolution of regional avalanche contribution in Western Canada and USA. *Projected changes of glacier area (a), avalanche contribution to accumulation (b) and total snow accumulation (c) for Western Canada and the USA. The black line corresponds to the historical period over which the mass balance model was calibrated using W5E5v2.0 data. The colored lines show the median future projections for different SSP scenarios, and the shaded areas indicate the 25th-75th percentile range. The different curves were smoothed using a 5-year rolling mean, except for the historical accumulation.*

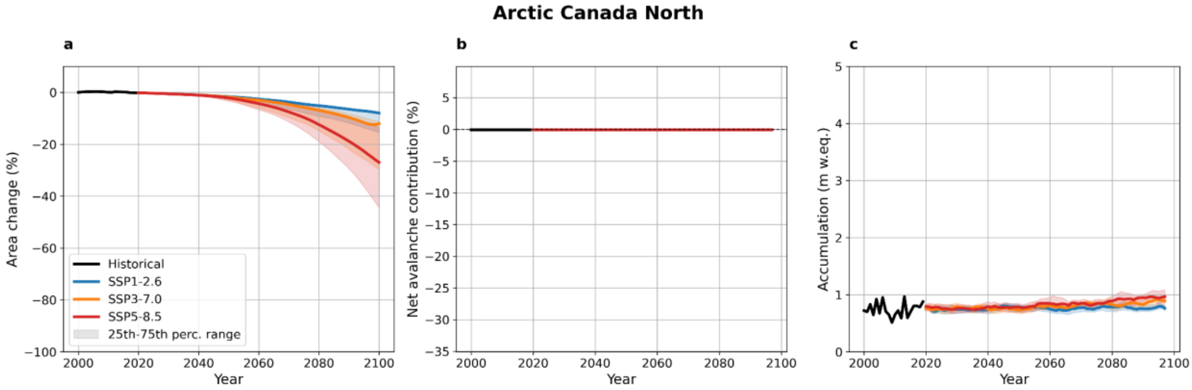

Supplementary Figure 28: Future evolution of regional avalanche contribution in Arctic Canada North. *Projected changes of glacier area (a), avalanche contribution to accumulation (b) and total snow accumulation (c) for Arctic Canada North. The black line corresponds to the historical period over which the mass balance model was calibrated using W5E5v2.0 data. The colored lines show the median future projections for different SSP scenarios, and the shaded areas indicate the 25th-75th percentile range. The different curves were smoothed using a 5-year rolling mean, except for the historical accumulation.*

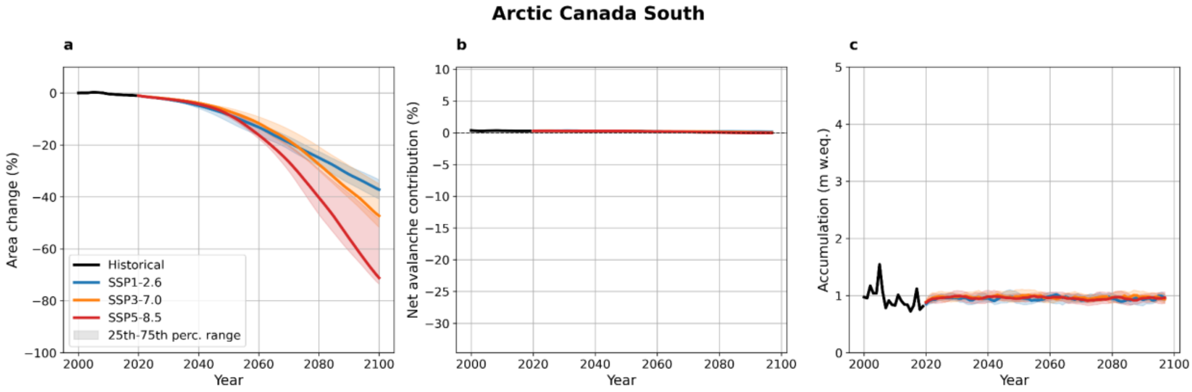

Supplementary Figure 29: Future evolution of regional avalanche contribution in Arctic Canada South. *Projected changes of glacier area (a), avalanche contribution to accumulation (b) and total snow accumulation (c) for Arctic Canada South. The black line corresponds to the historical period over which the mass balance model was calibrated using W5E5v2.0 data. The colored lines show the median future projections for different SSP scenarios, and the shaded areas indicate the 25th-75th percentile range. The different curves were smoothed using a 5-year rolling mean, except for the historical accumulation.*

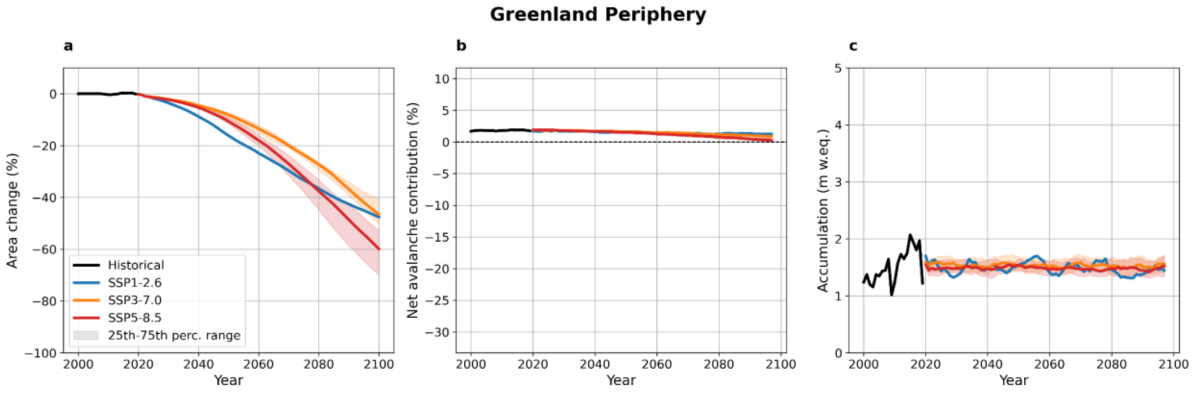

Supplementary Figure 30: Future evolution of regional avalanche contribution in the Greenland Periphery. *Projected changes of glacier area (a), avalanche contribution to accumulation (b) and total snow accumulation (c) for the Greenland Periphery. The black line corresponds to the historical period over which the mass balance model was calibrated using W5E5v2.0 data. The colored lines show the median future projections for different SSP scenarios, and the shaded areas indicate the 25th-75th percentile range. The different curves were smoothed using a 5-year rolling mean, except for the historical accumulation.*

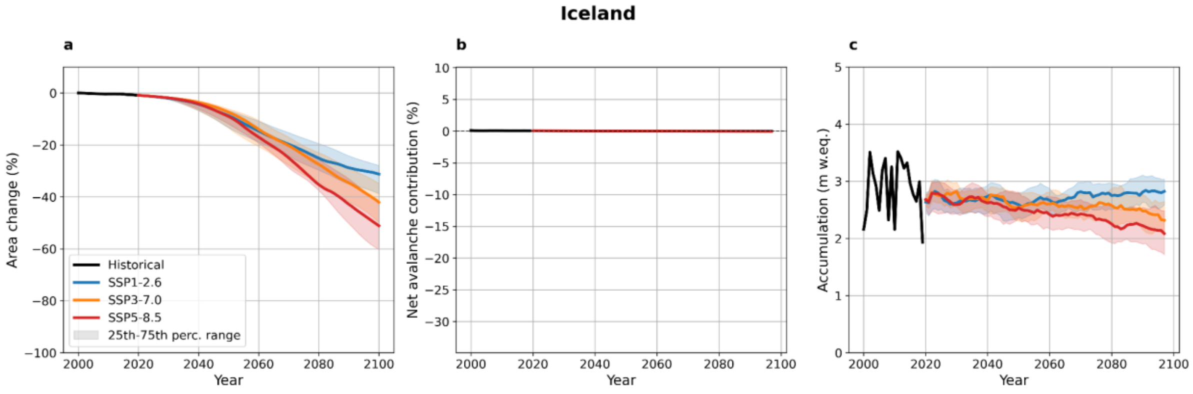

Supplementary Figure 31: Future evolution of regional avalanche contribution in Iceland. *Projected changes of glacier area (a), avalanche contribution to accumulation (b) and total snow accumulation (c) for Iceland. The black line corresponds to the historical period over which the mass balance model was calibrated using W5E5v2.0 data. The colored lines show the median future projections for different SSP scenarios, and the shaded areas indicate the 25th-75th percentile range. The different curves were smoothed using a 5-year rolling mean, except for the historical accumulation.*

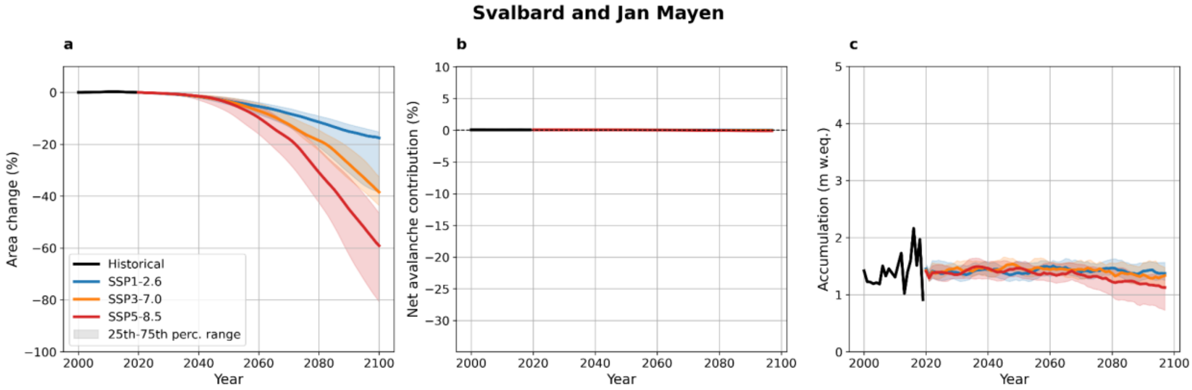

Supplementary Figure 32: Future evolution of regional avalanche contribution in Svalbard and Jan Mayen. *Projected changes of glacier area (a), avalanche contribution to accumulation (b) and total snow accumulation (c) for Svalbard and Jan Mayen. The black line corresponds to the historical period over which the mass balance model was calibrated using W5E5v2.0 data. The colored lines show the median future projections for different SSP scenarios, and the shaded areas indicate the 25th-75th percentile range. The different curves were smoothed using a 5-year rolling mean, except for the historical accumulation.*

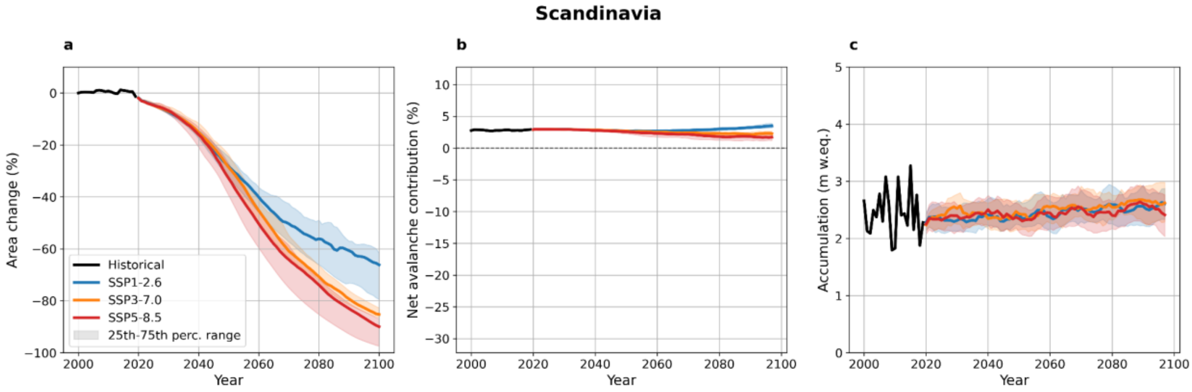

Supplementary Figure 33: Future evolution of regional avalanche contribution in Scandinavia. *Projected changes of glacier area (a), avalanche contribution to accumulation (b) and total snow accumulation (c) for Scandinavia. The black line corresponds to the historical period over which the mass balance model was calibrated using W5E5v2.0 data. The colored lines show the median future projections for different SSP scenarios, and the shaded areas indicate the 25th-75th percentile range. The different curves were smoothed using a 5-year rolling mean, except for the historical accumulation.*

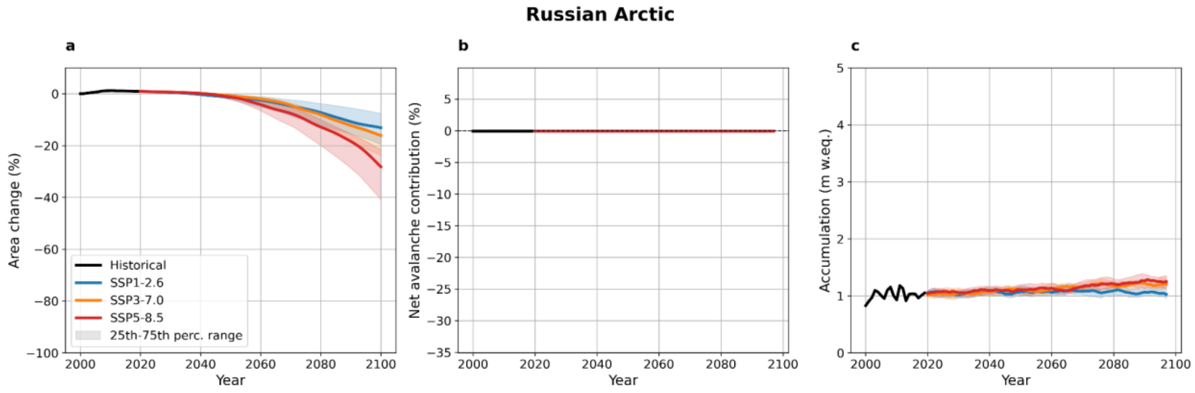

Supplementary Figure 34: Future evolution of regional avalanche contribution in the Russian Arctic. *Projected changes of glacier area (a), avalanche contribution to accumulation (b) and total snow accumulation (c) for the Russian Arctic. The black line corresponds to the historical period over which the mass balance model was calibrated using W5E5v2.0 data. The colored lines show the median future projections for different SSP scenarios, and the shaded areas indicate the 25th-75th percentile range. The different curves were smoothed using a 5-year rolling mean, except for the historical accumulation.*

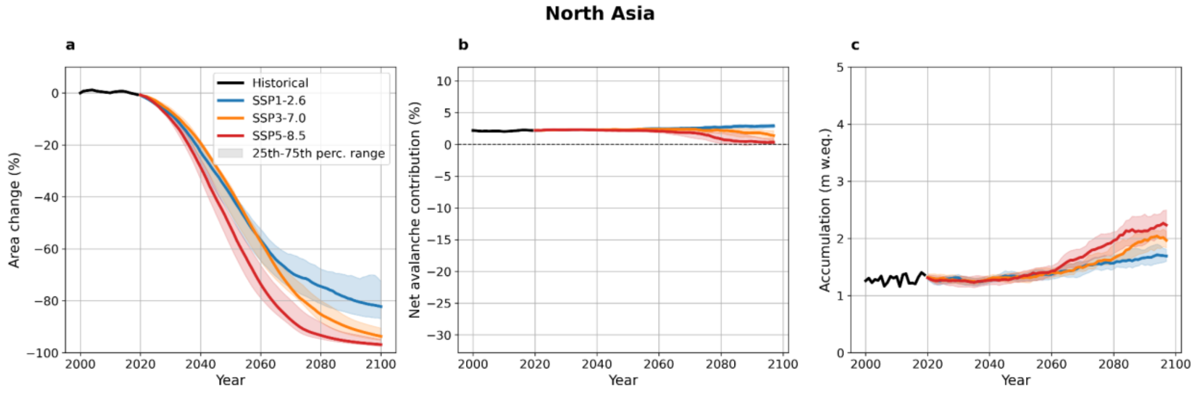

Supplementary Figure 35: Future evolution of regional avalanche contribution in North Asia. *Projected changes of glacier area (a), avalanche contribution to accumulation (b) and total snow accumulation (c) for North Asia. The black line corresponds to the historical period over which the mass balance model was calibrated using W5E5v2.0 data. The colored lines show the median future projections for different SSP scenarios, and the shaded areas indicate the 25th-75th percentile range. The different curves were smoothed using a 5-year rolling mean, except for the historical accumulation.*

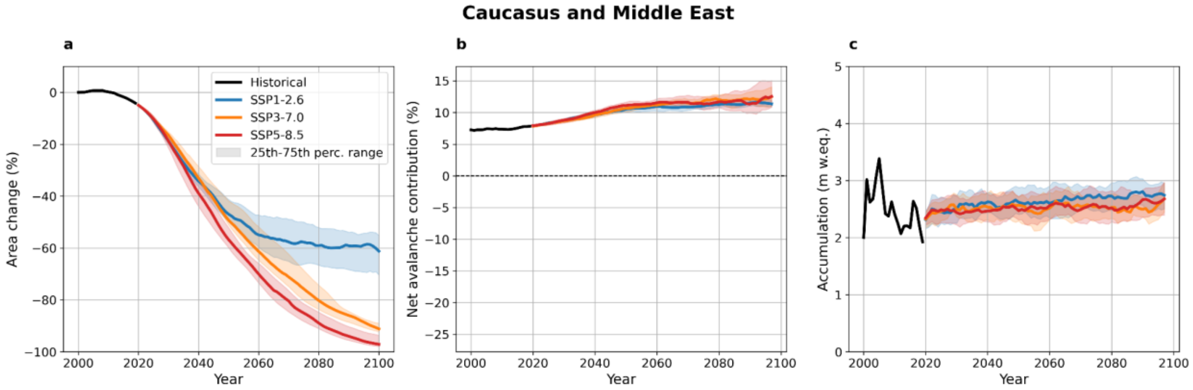

Supplementary Figure 36: Future evolution of regional avalanche contribution in Caucasus and the Middle East. *Projected changes of glacier area (a), avalanche contribution to accumulation (b) and total snow accumulation (c) for the Caucasus and Middle East. The black line corresponds to the historical period over which the mass balance model was calibrated using W5E5v2.0 data. The colored lines show the median future projections for different SSP scenarios, and the shaded areas indicate the 25th-75th percentile range. The different curves were smoothed using a 5-year rolling mean, except for the historical accumulation.*

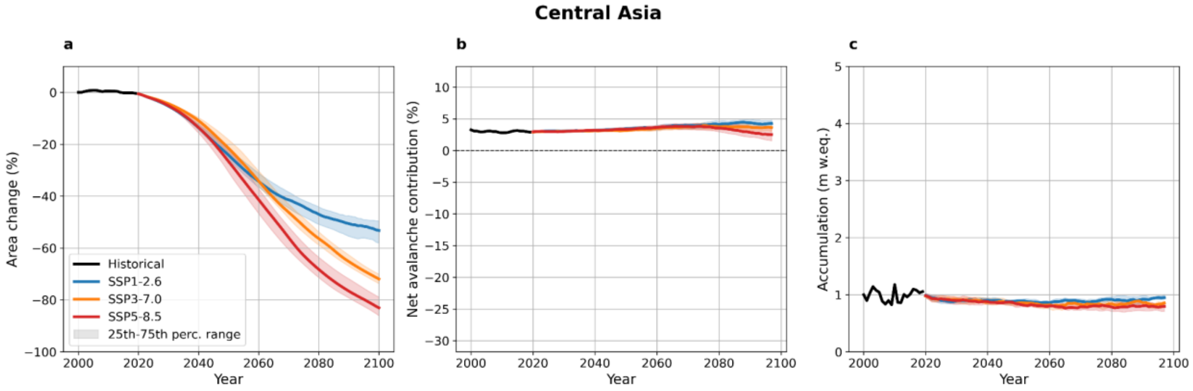

Supplementary Figure 37: Future evolution of regional avalanche contribution in Central Asia. *Projected changes of glacier area (a), avalanche contribution to accumulation (b) and total snow accumulation (c) for Central Asia. The black line corresponds to the historical period over which the mass balance model was calibrated using W5E5v2.0 data. The colored lines show the median future projections for different SSP scenarios, and the shaded areas indicate the 25th-75th percentile range. The different curves were smoothed using a 5-year rolling mean, except for the historical accumulation.*

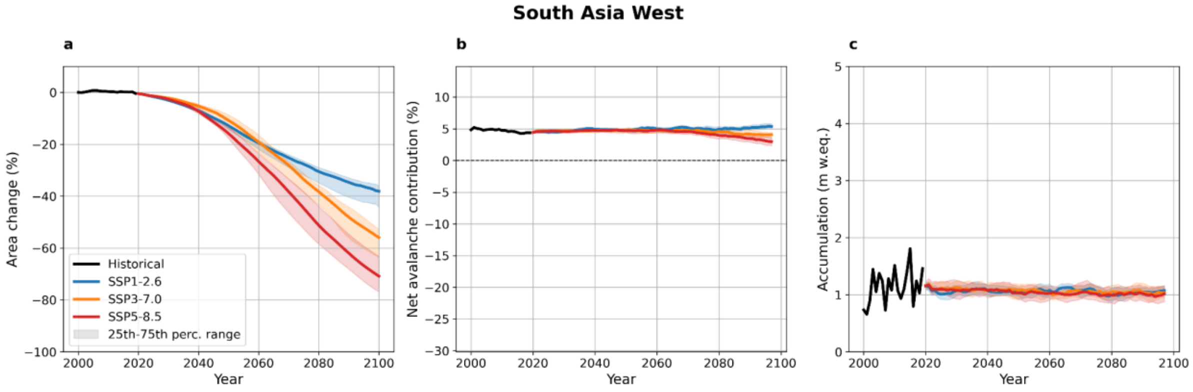

Supplementary Figure 38: Future evolution of regional avalanche contribution in South Asia West. *Projected changes of glacier area (a), avalanche contribution to accumulation (b) and total snow accumulation (c) for South Asia West. The black line corresponds to the historical period over which the mass balance model was calibrated using W5E5v2.0 data. The colored lines show the median future projections for different SSP scenarios, and the shaded areas indicate the 25th-75th percentile range. The different curves were smoothed using a 5-year rolling mean, except for the historical accumulation.*

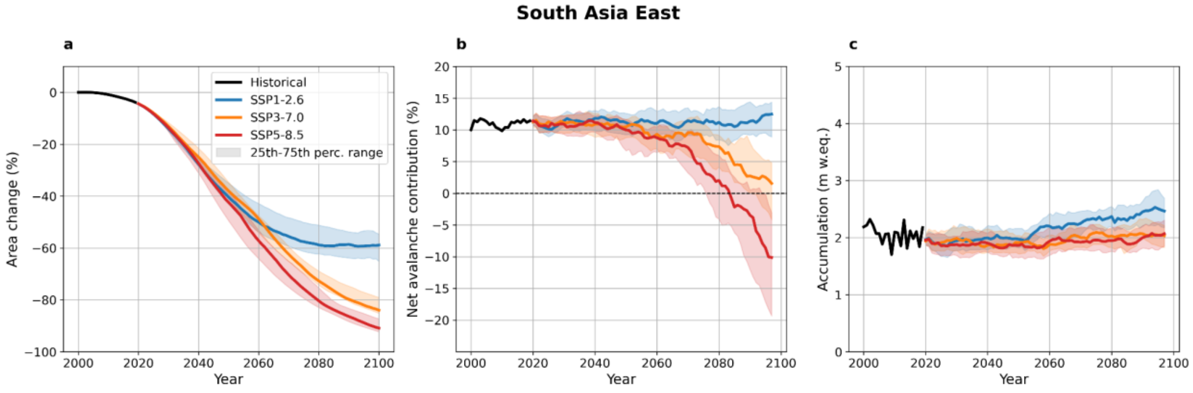

Supplementary Figure 39: Future evolution of regional avalanche contribution in South Asia East. *Projected changes of glacier area (a), avalanche contribution to accumulation (b) and total snow accumulation (c) for South Asia East. The black line corresponds to the historical period over which the mass balance model was calibrated using W5E5v2.0 data. The colored lines show the median future projections for different SSP scenarios, and the shaded areas indicate the 25th-75th percentile range. The different curves were smoothed using a 5-year rolling mean, except for the historical accumulation.*

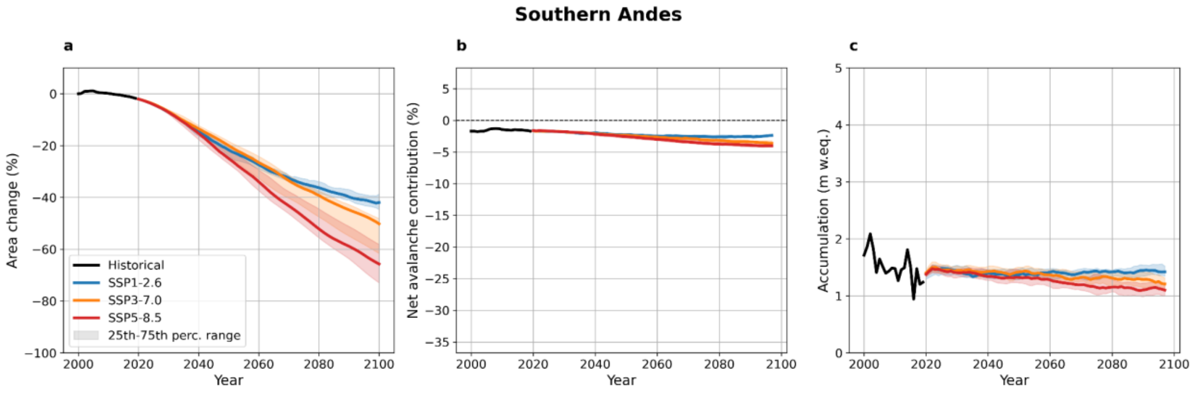

Supplementary Figure 40: Future evolution of regional avalanche contribution in the Southern Andes. *Projected changes of glacier area (a), avalanche contribution to accumulation (b) and total snow accumulation (c) for the Southern Andes. The black line corresponds to the historical period over which the mass balance model was calibrated using W5E5v2.0 data. The colored lines show the median future projections for different SSP scenarios, and the shaded areas indicate the 25th-75th percentile range. The different curves were smoothed using a 5-year rolling mean, except for the historical accumulation.*

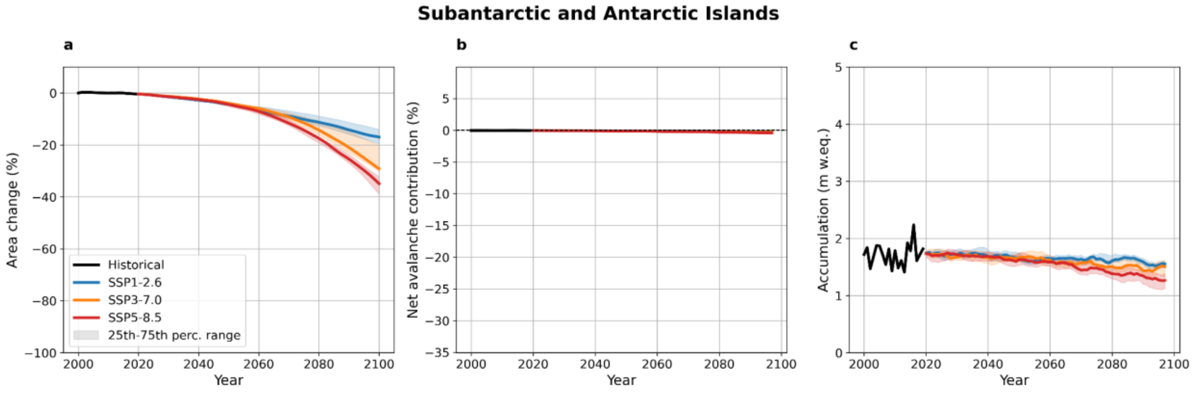

Supplementary Figure 41: Future evolution of regional avalanche contribution in the Subantarctic and Antarctic Islands. *Projected changes of glacier area (a), avalanche contribution to accumulation (b) and total snow accumulation (c) for the Subantarctic and Antarctic Islands. The black line corresponds to the historical period over which the mass balance model was calibrated using W5E5v2.0 data. The colored lines show the median future projections for different SSP scenarios, and the shaded areas indicate the 25th-75th percentile range. The different curves were smoothed using a 5-year rolling mean, except for the historical accumulation.*

Influence of temporarily updating the avalanche contribution

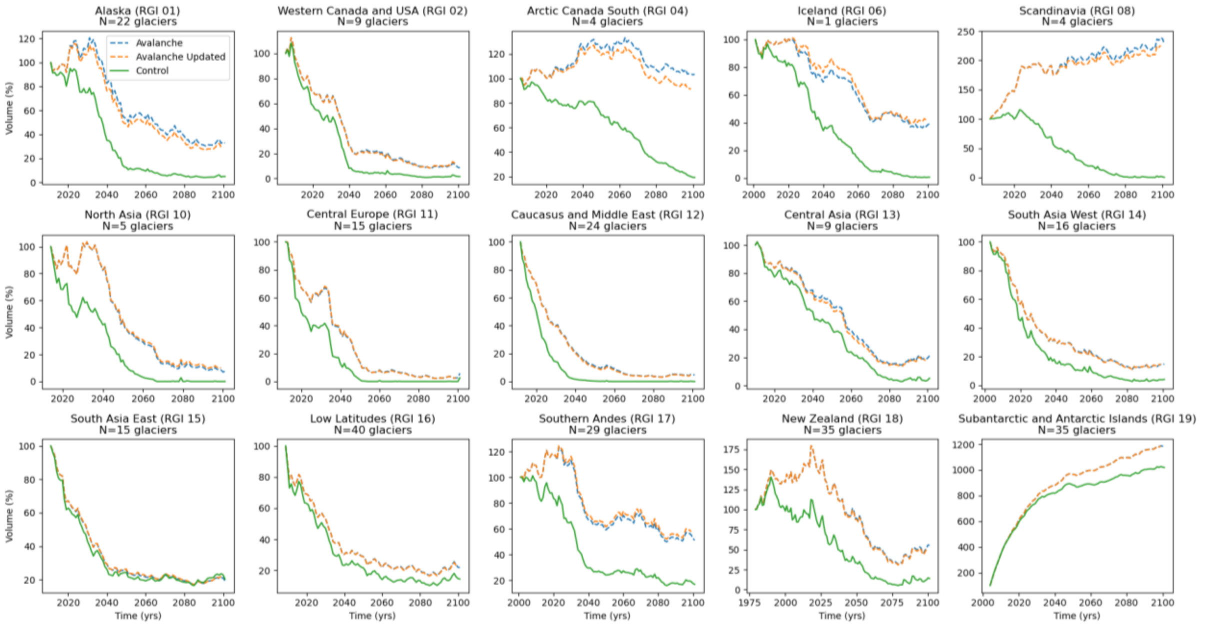

***Supplementary Figure 42: Influence of temporarily updating the avalanche contribution on glaciers with a negative avalanche contribution**. Glacier volume changes for 263 randomly selected glaciers with a glacier-wide avalanche correction factor ($k_{ava, gl}$) lower than 0.8 in 15 RGI regions for the ipsl-cm6a-lr_r1i1p1f1 GCM under the SSP 1-2.6 scenario. The green line indicates the scenario without avalanches, the blue dashed line corresponds to the scenario with fixed avalanche contribution calculated over the period 2020-2100, and the orange line corresponds to the scenario with an avalanche contribution updated every 20 years.*

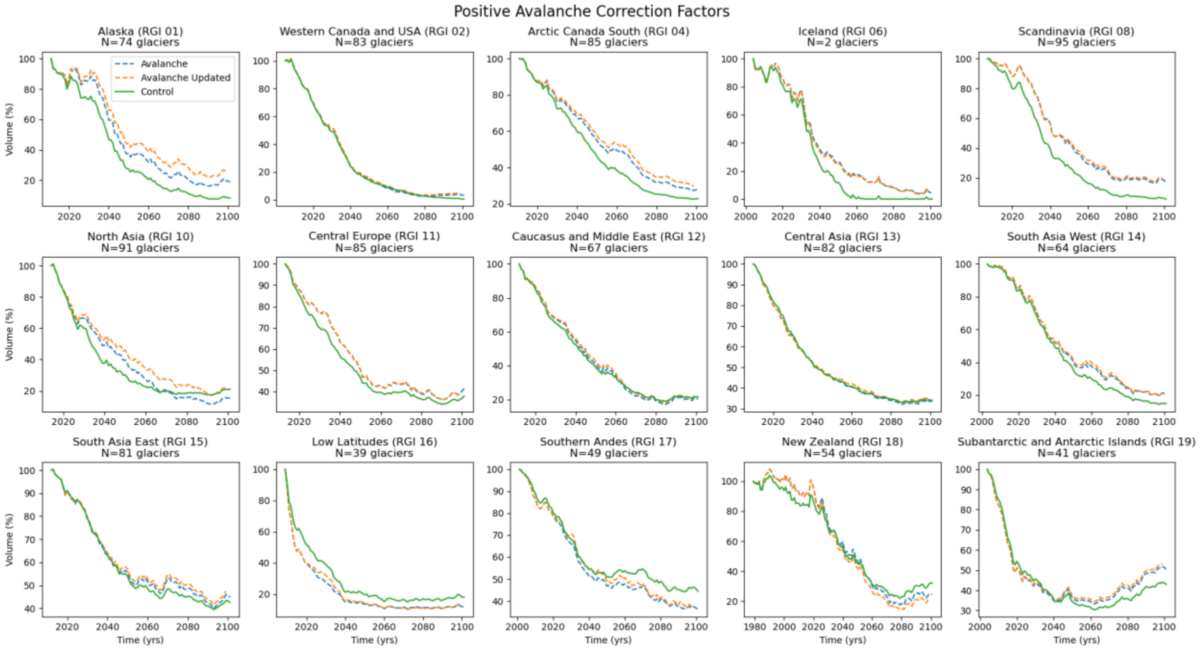

Supplementary Figure 43: **Influence of temporarily updating the avalanche contribution on glaciers with a positive avalanche contribution**. *Glacier volume changes for 1003 randomly selected glaciers with a glacier-wide avalanche correction factor (*$k_{ava,\ gl}$*) higher than 1.2 in 15 RGI regions for the ipsl-cm6a-lr_r1i1p1f1 GCM under the SSP 1-2.6 scenario. The green line indicates the scenario without avalanches, the blue dashed line corresponds to the scenario with fixed avalanche contribution calculated over the period 2020-2100, and the orange line corresponds to the scenario with an avalanche contribution updated every 20 years.*

Influence of DEM spatial resolution

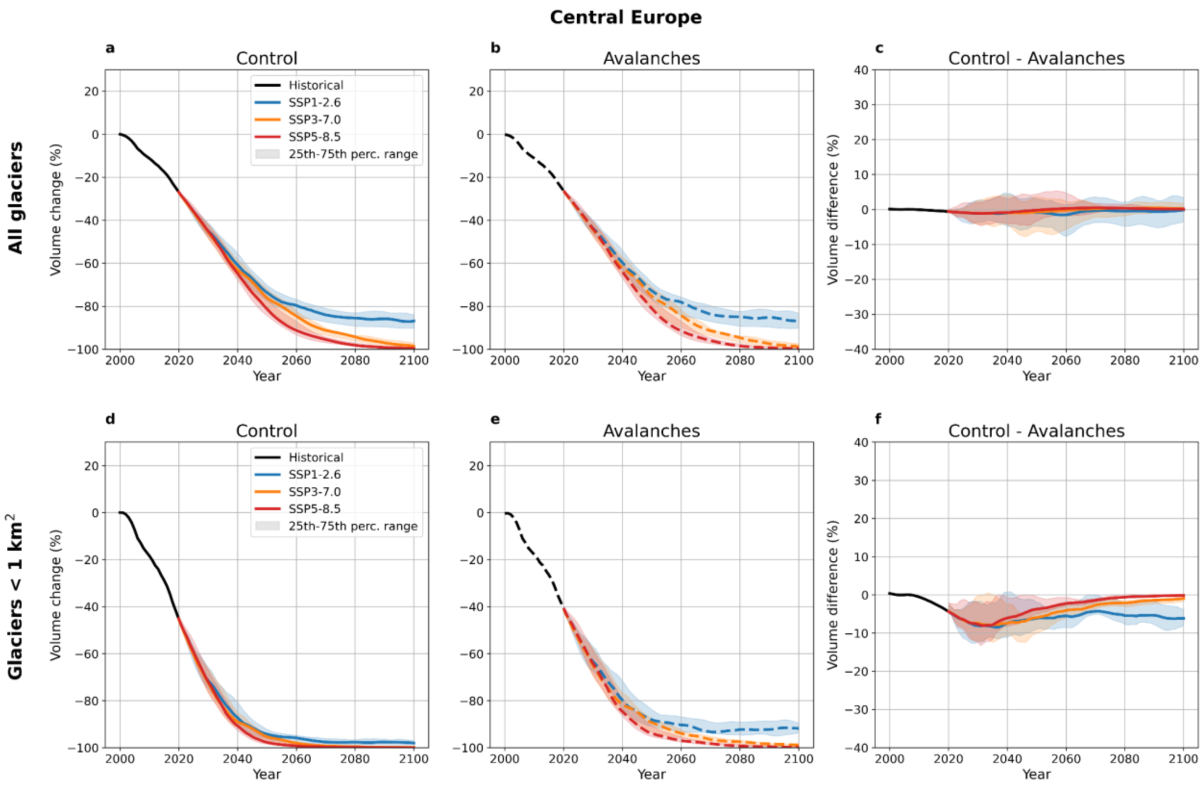

Supplementary Figure 44: Influence of avalanches on regional glacier volume changes in Central Europe at 50 m resolution. *Projected volume changes of all glaciers (**a-c**) and only the glaciers smaller than 1 km^2 (**d-f**) in Central Europe, from the 'Control' (**a, d**) and 'Avalanches' (**b, e**) simulations, using a fixed DEM resolution of 50 m. The right-hand plots (**c, f**) show the difference between the 'Control' and the 'Avalanches' simulations, so that negative values indicate more volume in the 'Avalanches' simulations. All percentages are given relative to the initial volume in 2000 in the 'Control' simulation. The black line corresponds to the historical period over which the mass balance model was calibrated using W5E5v2.0 data. The colored lines show the median future projections for different SSP scenarios, and the shaded areas indicate the 25th-75th percentile range. The different curves were smoothed using a 5-year rolling mean.*

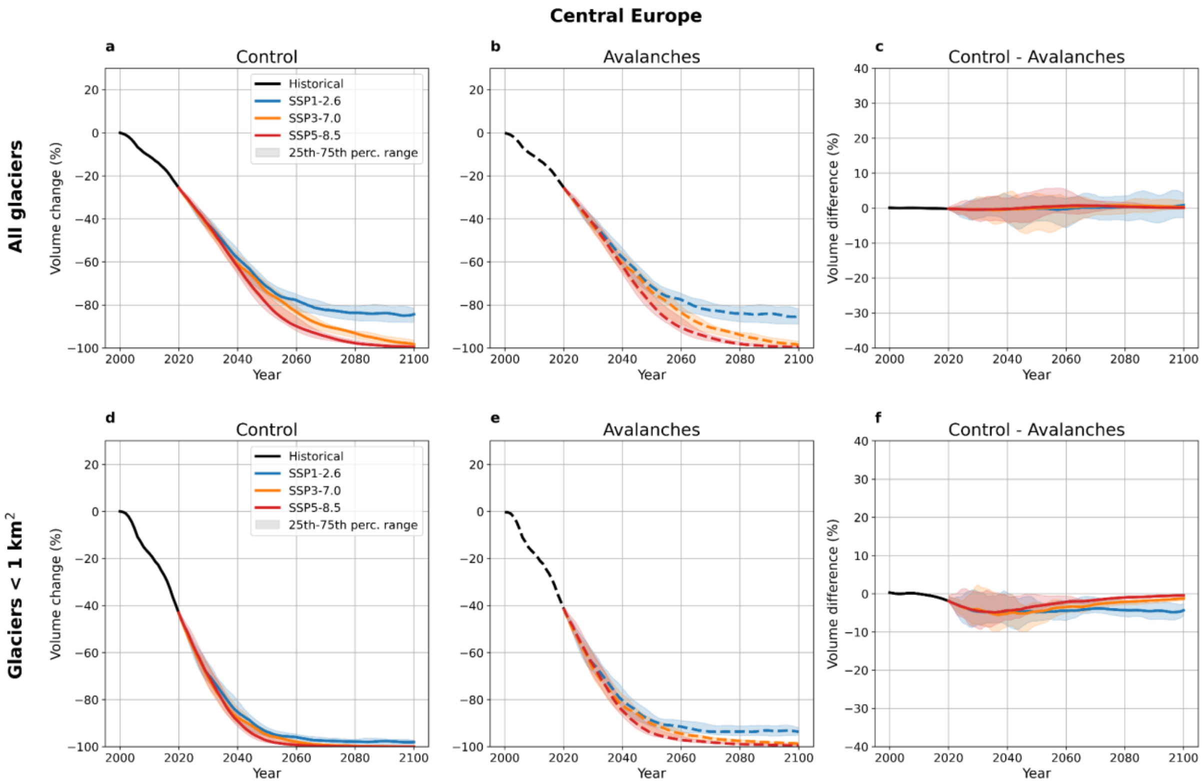

Supplementary Figure 45: Influence of avalanches on regional glacier volume changes in Central Europe at 100 m resolution. *Projected volume changes of all glaciers (**a-c**) and only the glaciers smaller than 1 km^2 (**d-f**) in Central Europe, from the 'Control' (**a, d**) and 'Avalanches' (**b, e**) simulations, using a fixed DEM resolution of 100 m. The right-hand plots (**c, f**) show the difference between the 'Control' and the 'Avalanches' simulations, so that negative values indicate more volume in the 'Avalanches' simulations. All percentages are given relative to the initial volume in 2000 in the 'Control' simulation. The black line corresponds to the historical period over which the mass balance model was calibrated using W5E5v2.0 data. The colored lines show the median future projections for different SSP scenarios, and the shaded areas indicate the 25th-75th percentile range. The different curves were smoothed using a 5-year rolling mean.*

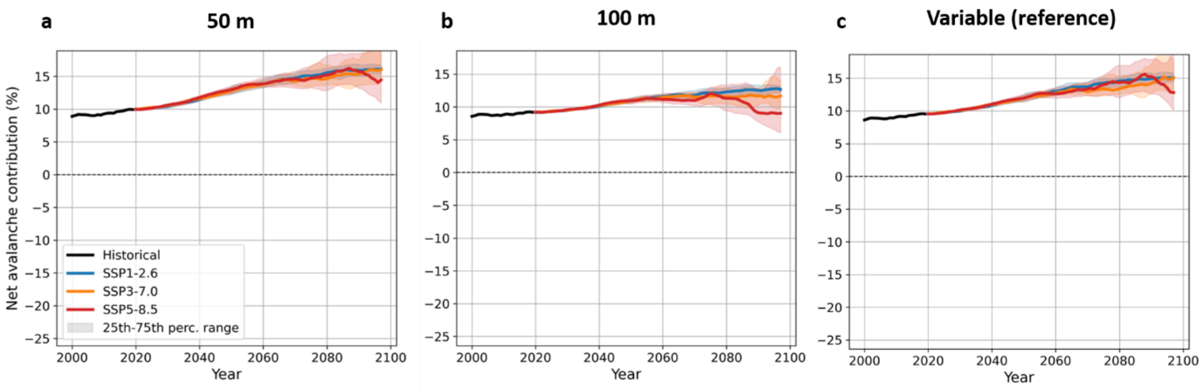

Supplementary Figure 46: Influence of DEM spatial resolution on projected avalanche contribution. *Projected changes of avalanche contribution to glacier accumulation for Central*

*Europe at 50 m (**a**), 100 m (**b**) and at the original variable resolution (**c**). The black line corresponds to the historical period over which the mass balance model was calibrated using W5E5v2.0 data. The colored lines show the median future projections for different SSP scenarios, and the shaded areas indicate the 25th-75th percentile range. The different curves were smoothed using a 5-year rolling mean.*

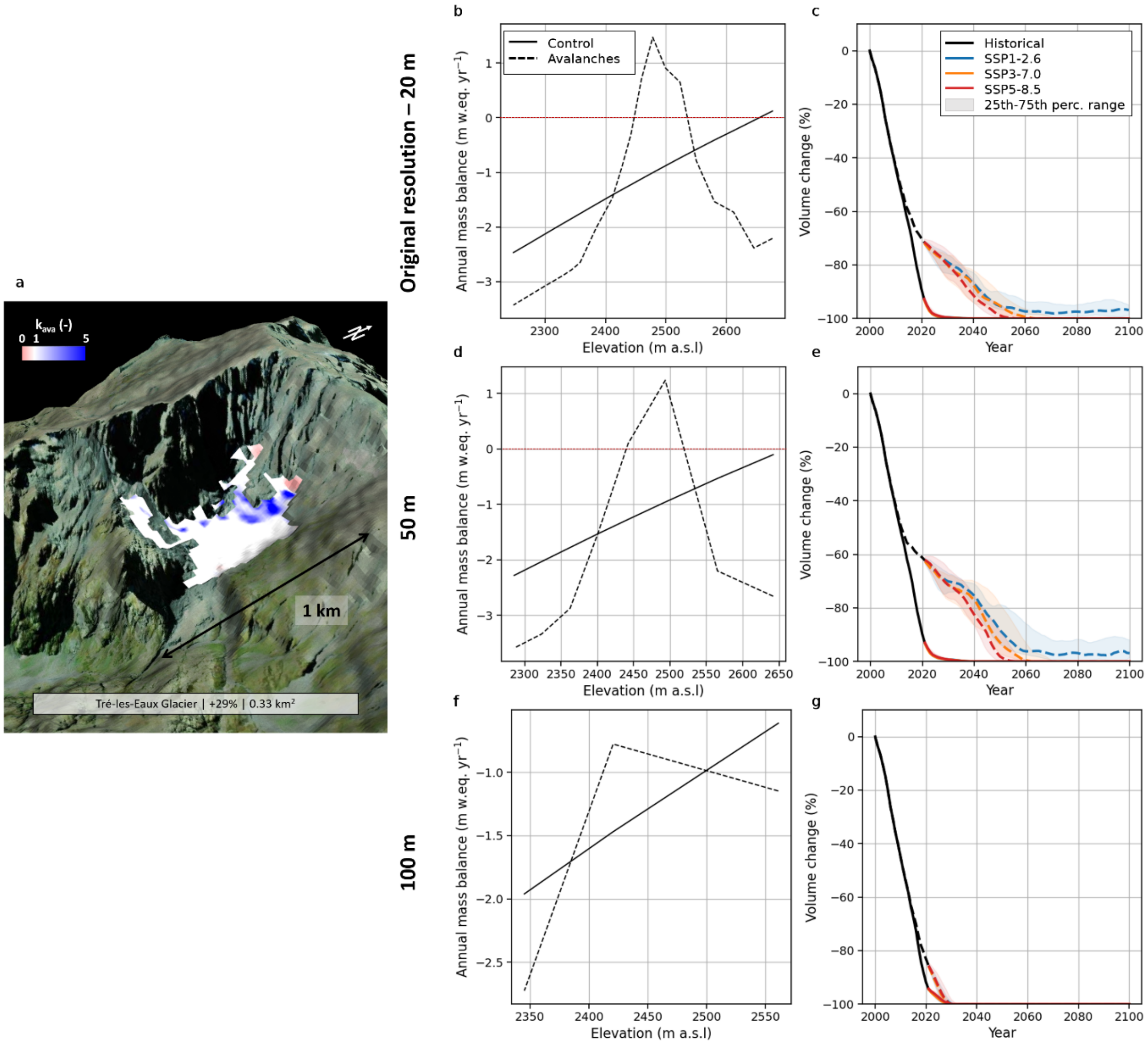

***Supplementary Figure 47: Influence of DEM spatial resolution on volume change of Tré-les-Eaux Glacier. a** Tré-les-Eaux Glacier in Central Europe with altitudinal mass balance and 21st century volume changes **b-c** at the original 20 m resolution, **d-e** at 50 m resolution and **f-g** at 100 m resolution. The left panel shows a 3D view of the glacier with the average avalanche correction factor (k_{ava}) over the period 01/2000-12/2019 indicated by the red-white-blue shade wrapped over the glacier extents from the Randolph Glacier Inventory (RGI) 6.0. The background topography is from the DEM used to run the OGGM simulations. The numbers indicate the glacier-wide avalanche contribution and the glacier area. The central panels show the altitudinal mass balance for the 'Control' and 'Avalanches' simulations, with the red dashed line corresponding to a mass balance value of 0 m w.eq. yr^{-1}. The right panels show the past*

and future volume changes of these glaciers as modelled using OGGM. All percentages are given relative to the initial glacier volume in 2000 in the Control scenario. The black line corresponds to the historical period over which the mass balance model was calibrated using W5E5v2.0 data. The colored lines show the median future projections for different SSP scenarios, and the shaded areas indicate the 25th-75th percentile range. The different curves were smoothed using a 5-year rolling mean.

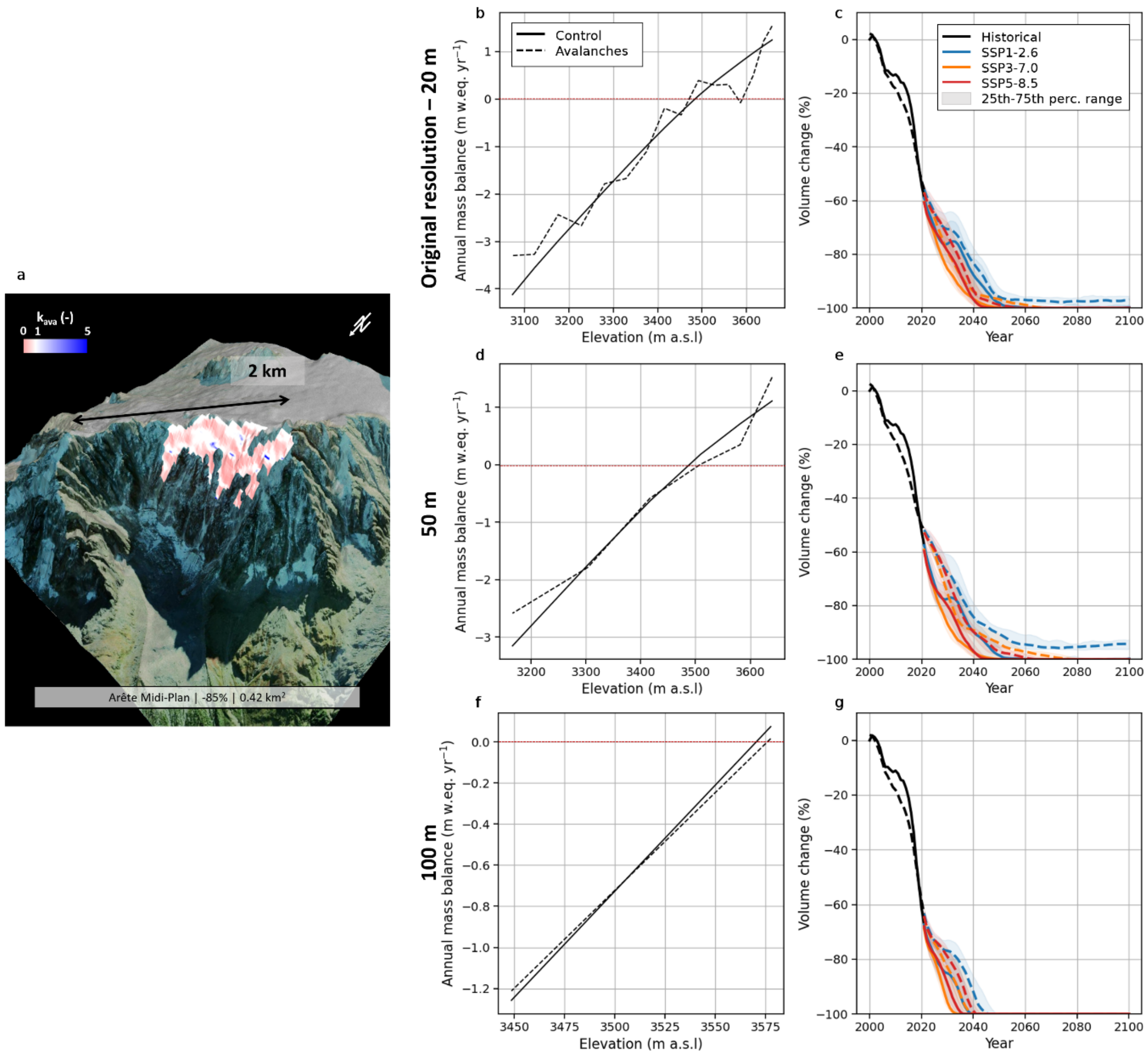

***Supplementary Figure 48: Influence of DEM spatial resolution on volume change of Arête Midi-Plan. a** Arête Midi-Plan in Central Europe with altitudinal mass balance and 21st century volume changes **b-c** at the original 20 m resolution, **d-e** at 50 m resolution and **f-g** at 100 m resolution. The left panel shows a 3D view of the glacier with the average avalanche correction factor (k_{ava}) over the period 01/2000-12/2019 indicated by the red-white-blue shade wrapped over the glacier extents from the Randolph Glacier Inventory (RGI) 6.0. The background topography is from the DEM used to run the OGGM simulations. The numbers indicate the glacier-wide avalanche contribution and the glacier area. The central panels show the altitudinal mass balance for the 'Control' and 'Avalanches' simulations, with the red dashed*

line corresponding to a mass balance value of 0 m w.eq. yr^{-1}. The right panels show the past and future volume changes of these glaciers as modelled using OGGM. All percentages are given relative to the initial glacier volume in 2000 in the Control scenario. The black line corresponds to the historical period over which the mass balance model was calibrated using W5E5v2.0 data. The colored lines show the median future projections for different SSP scenarios, and the shaded areas indicate the 25th-75th percentile range. The different curves were smoothed using a 5-year rolling mean.

Evaluation against Sentinel-1 avalanche deposits

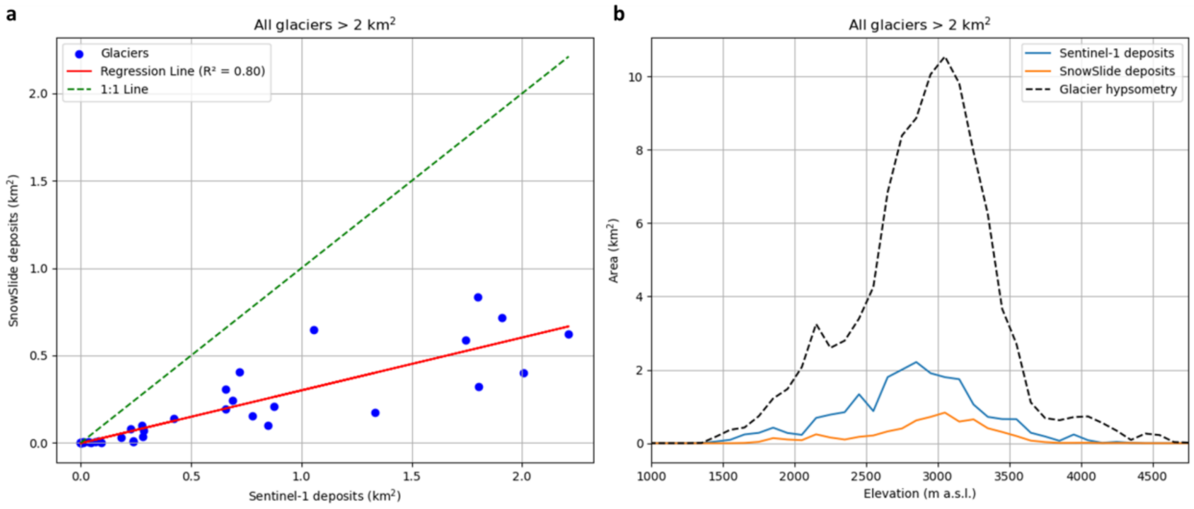

Supplementary Figure 49: Comparison of the SnowSlide and Sentinel-1 avalanche deposit extents over the glaciers larger than 2 km^2 in the Mt Blanc region*. *a* *Area of SnowSlide and Sentinel-1 deposits for each individual glacier.* ***b *Hypsometry of the SnowSlide and Sentinel-1 avalanche deposits and glaciers.*

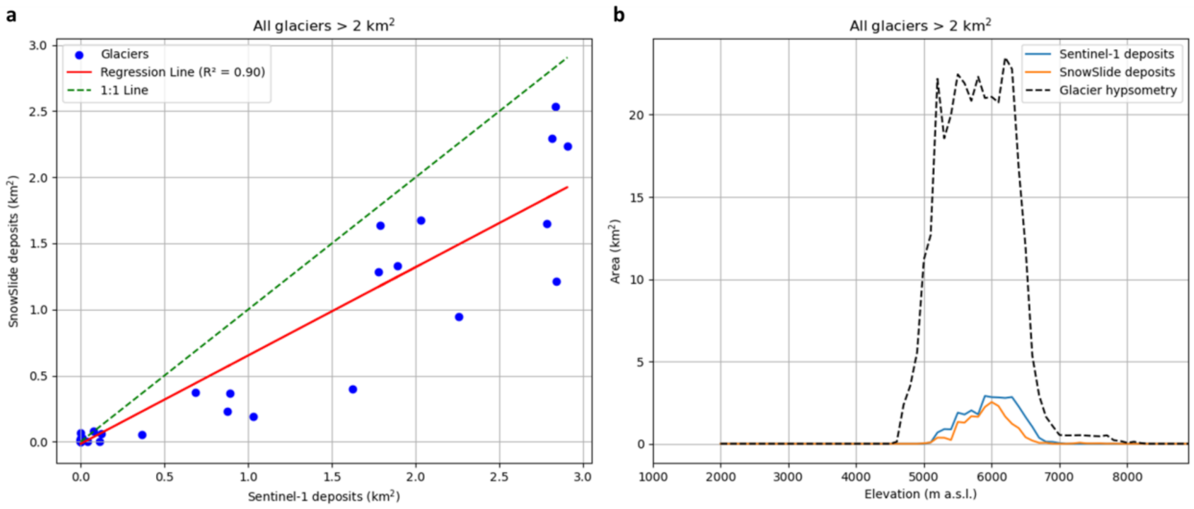

Supplementary Figure 50: Comparison of the SnowSlide and Sentinel-1 avalanche deposit extents over the glaciers larger than 2 km² in the Everest region. a *Area of SnowSlide and Sentinel-1 deposits for each individual glacier.* ***b*** *Hypsometry of the SnowSlide and Sentinel-1 avalanche deposits and glaciers.*

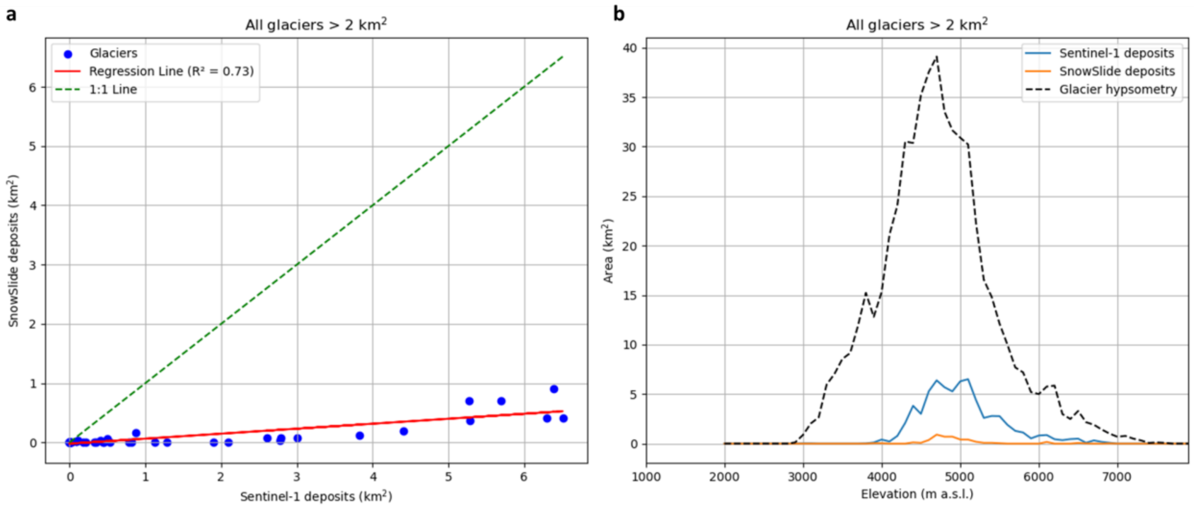

Supplementary Figure 51: Comparison of the SnowSlide and Sentinel-1 avalanche deposit extents over the glaciers larger than 2 km² in the Hispar region. a *Area of SnowSlide and Sentinel-1 deposits for each individual glacier.* ***b*** *Hypsometry of the SnowSlide and Sentinel-1 avalanche deposits and glaciers.*

Evaluation against mass balance measurements

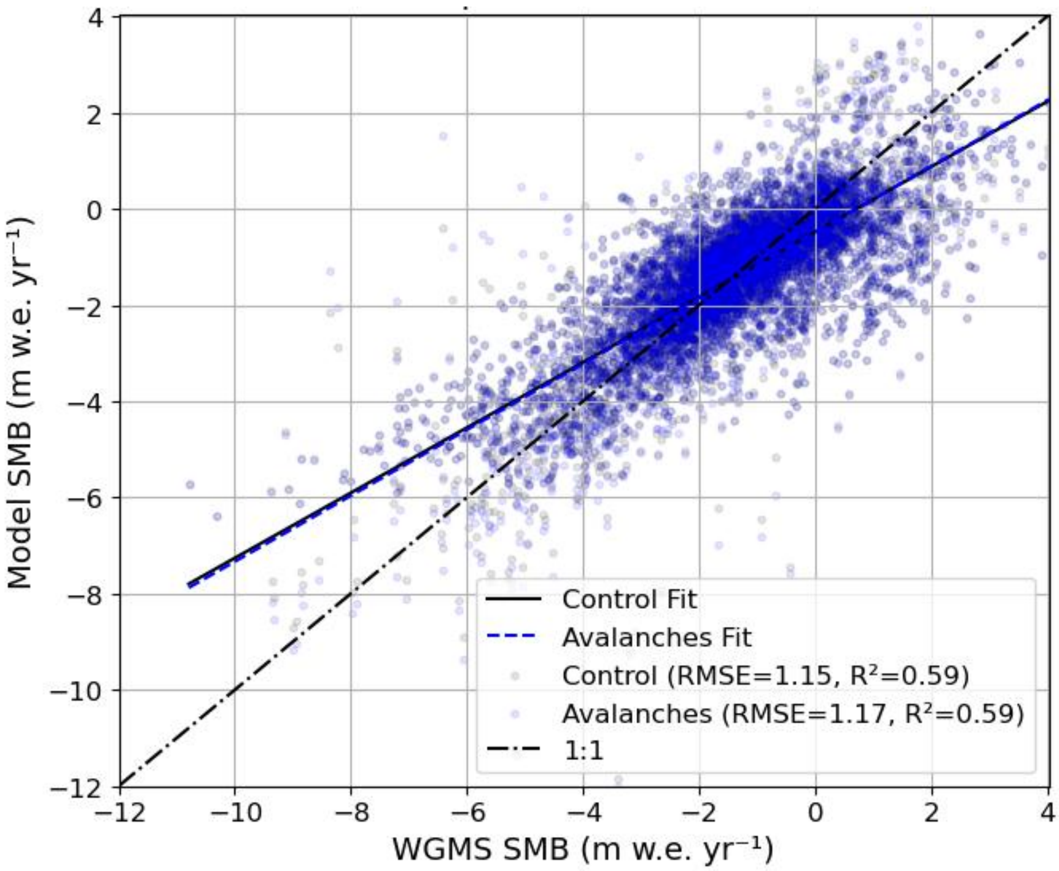

Supplementary Figure 52: Comparison of the modeled and measured annual point mass balance from the World Glacier Monitoring Service. *The observation period goes from 2000 to 2020. The results from the Control simulations are in black and from the Avalanches simulations in blue.*

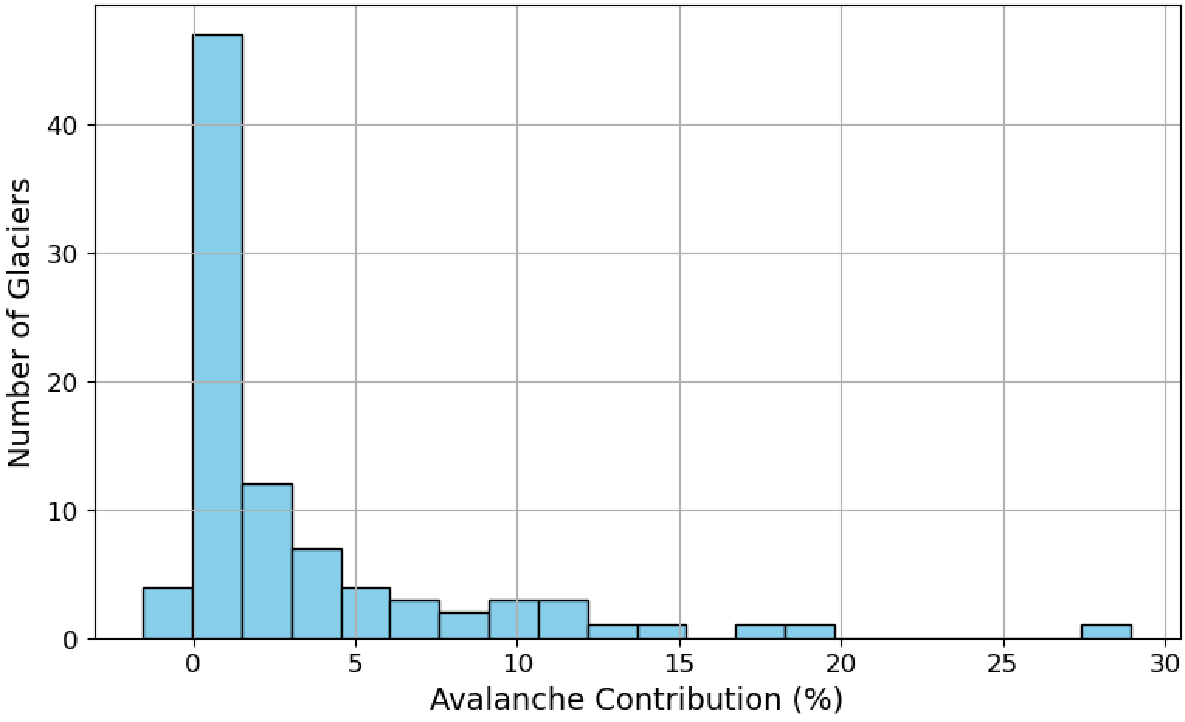

Supplementary Figure 53: Avalanche contribution of WGMS glaciers. *Histogram of the WGMS glaciers with at least one annual mass balance measurement in the period 2000-2020 as a function of their glacier-wide avalanche contribution.*

References

1. RGI Consortium. Randolph Glacier Inventory – A Dataset of Global Glacier Outlines: Version 6.0: Technical Report, Global Land Ice Measurements from Space, Colorado, USA. Digital Media. https://doi.org/https://doi.org/10.7265/N5-RGI-60 (2017) doi:https://doi.org/10.7265/N5-RGI-60.
